# The Mass-Longevity Triangle: Pareto Optimality and the Geometry of Life-History Trait Space

**DOI:** 10.1371/journal.pcbi.1004524

**Published:** 2015-10-14

**Authors:** Pablo Szekely, Yael Korem, Uri Moran, Avi Mayo, Uri Alon

**Affiliations:** 1 Department of Molecular Cell Biology, The Weizmann Institute of Science, Rehovot, Israel; 2 Department of Plant Science, The Weizmann Institute of Science, Rehovot, Israel; University of Tokyo, TOKYO

## Abstract

When organisms need to perform multiple tasks they face a fundamental tradeoff: no phenotype can be optimal at all tasks. This situation was recently analyzed using Pareto optimality, showing that tradeoffs between tasks lead to phenotypes distributed on low dimensional polygons in trait space. The vertices of these polygons are archetypes—phenotypes optimal at a single task. This theory was applied to examples from animal morphology and gene expression. Here we ask whether Pareto optimality theory can apply to life history traits, which include longevity, fecundity and mass. To comprehensively explore the geometry of life history trait space, we analyze a dataset of life history traits of 2105 endothermic species. We find that, to a first approximation, life history traits fall on a triangle in log-mass log-longevity space. The vertices of the triangle suggest three archetypal strategies, exemplified by bats, shrews and whales, with specialists near the vertices and generalists in the middle of the triangle. To a second approximation, the data lies in a tetrahedron, whose extra vertex above the mass-longevity triangle suggests a fourth strategy related to carnivory. Each animal species can thus be placed in a coordinate system according to its distance from the archetypes, which may be useful for genome-scale comparative studies of mammalian aging and other biological aspects. We further demonstrate that Pareto optimality can explain a range of previous studies which found animal and plant phenotypes which lie in triangles in trait space. This study demonstrates the applicability of multi-objective optimization principles to understand life history traits and to infer archetypal strategies that suggest why some mammalian species live much longer than others of similar mass.

## Author Summary

Understanding why some mammals live longer than others is of crucial interest. Here we study how longevity relates to other life-history traits, using data on about 2000 species of mammals and birds. In contrast to the tradition in which traits fall on a line in logarithmic coordinates, called allometric lines. We find that mass and longevity of mammals and birds fills out a triangle in logarithmic axes, not a line. Thus, mammals of low and intermediate mass can have a wide range of life spans. We interpret this geometry using Pareto optimality theory: The triangle suggests three different life strategies or tasks. Animals near the vertices of the triangle are specialists at one of the strategies, animals near the center are generalists. Analyzing the data at higher resolution suggests an additional fourth strategy related to carnivores. The mass-longevity triangle offers a new coordinate system for comparative studies of animal longevity.

## Introduction

Mammals can have very different lifespans, and it is of great interest to understand why longevity differs between species. Recent studies use comparative approaches to understand mechanisms for longevity in diverse mammalian species, especially species which are long lived [1–3]. In order to expand such studies to a wider range of species, a good understanding of how longevity varies with other traits, such as mass, is needed.

**Fig 1:**
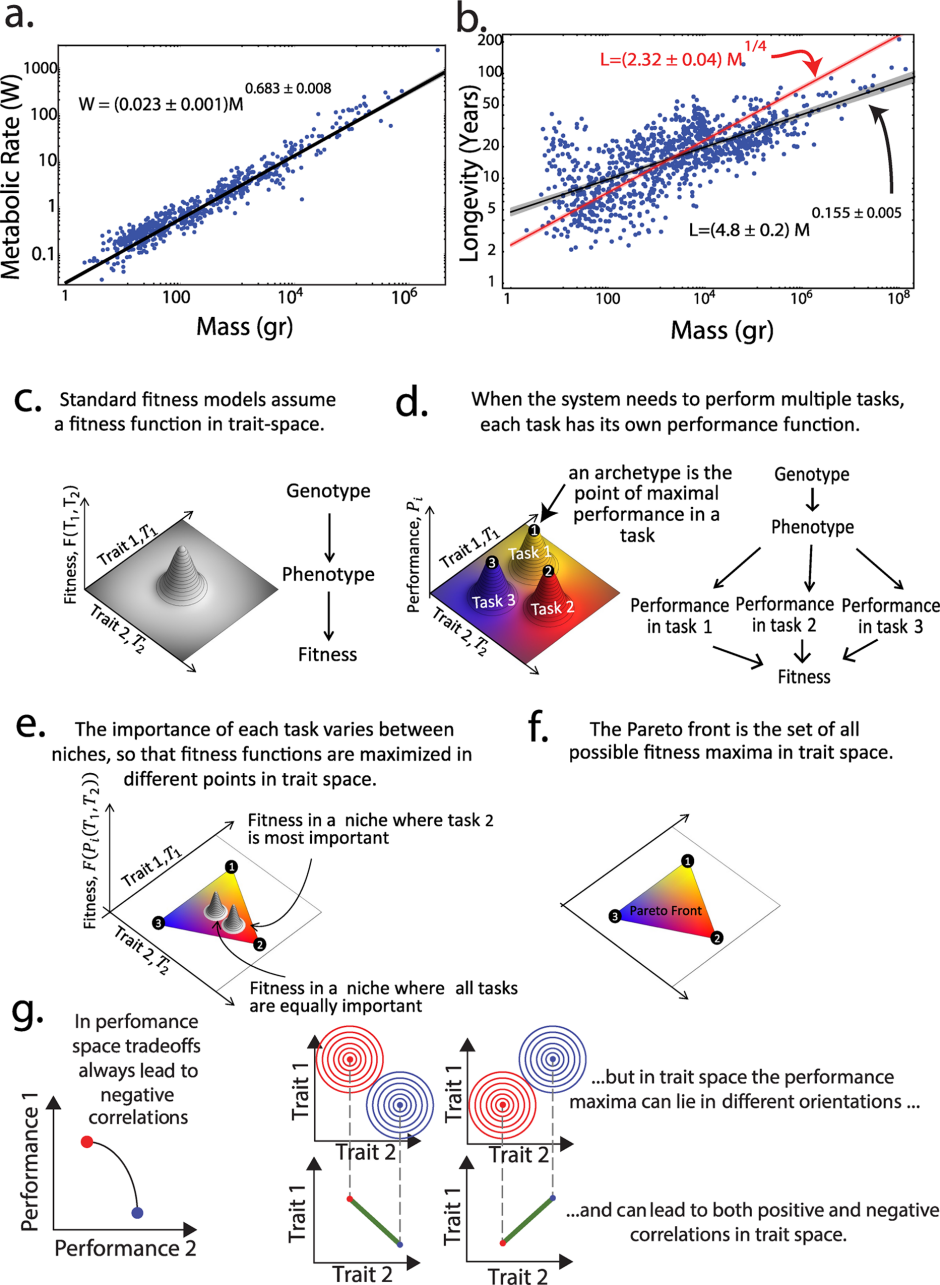
Tradeoffs lead to polygonal suites of variation according to Pareto theory. (a) Mammalian metabolic rate goes as a clean allometric line with respect to mass, known as Kleiber’s law (data from anAge [43]). (b) Mammalian longevity does not fall cleanly on a line with respect to mass. Shown are regressions to a power law and a 1/4 power law. Shaded areas represent 95% confidence intervals. (c) The fitness landscape is given by the fitness of each phenotype in trait space. (d) When fitness is composed of several tasks, one can draw a performance landscape for each task. The phenotype that maximizes performance at a task is called the archetype. (e) Organisms in niches in which one task is more important than the others have a fitness maximum close to the corresponding archetype. Generalists have a fitness maximum near the middle of the Pareto front. (f) The suite of all fitness maxima in all conceivable niches in which fitness increases with task performance is called the Pareto front. It is a polygon (or a slightly curved polygon [74]) whose vertices are the archetype. In the case of three tasks, the Pareto front is a triangle. (g) Positive correlation between traits can occur even if there is a tradeoff between their corresponding tasks. Stearns’ life history theory considers performance space, with tradeoffs defined as negative correlations between performances. Pareto theory concerns trait space, not performance space. The performance functions, shown here as contours, can have maxima (archetypes) in any spatial relationship leading to either positive or negative correlation in trait space, even though in both cases the curves stem from tradeoffs.

It is well known that larger animals live longer, and some authors suggest an allometric relation in which *L* ∼ *M* ^1/4^ [4–7]. Outliers to this relation have long been noted such as flying animals which are long lived given their mass [8,9], as well as arboreal species [10]. In contrast to well-known power-laws such as the ¾-power law relating mass and metabolic rate (Fig 1A), longevity and mass do not fall along a clean line in log space (Fig 1B), suggesting that a simple allometric law may not apply. In general, the allometric law explains only about 35% of the lifespan variation [11]. More recent work used multi-dimensional linear [12] regression to connect multiple traits such as brain size with longevity [13–17].This diversity of lifespan is of interest, since it may point to ways of understanding the regulation of longevity [2,3,18–21]. Life history traits such as longevity and reproductive parameters are closely linked with the ecological niche and environmental interactions of each species [22–26].

Short and long lifetimes have been associated with different global life-history strategies, namely rapid growth and reproduction versus long-term survival with slow reproduction. Work in the 1970s suggested a theory of r and K strategies based on the logistic equation[27–30], where r-strategists have rapid reproduction and short lifespan, and K-specialists have long lifetime and slow reproduction. More recent work has challenged r-K theory and the use of the logistic equation; Recent work empirically supports the existence of a continuum of life-history traits known as the fast–slow continuum[12,31,32,33]. A different approach, within Stearn’s life-history theory [34], focuses on physiological tradeoffs defined as negative correlations between traits which are hypothesized to result from competition over physiological resources.

Here, we approach the relation of mass, longevity and other traits by analyzing a large database of endotherm (mammal and bird) life-history traits. We use concepts from a recent theoretical advance by Shoval et al [35] in understanding suites of variations when organisms need to perform multiple tasks[36], based on Pareto optimality. A Pareto optimal system cannot perform better in all task, but rather in order to improve in a task it has to reduce its performance in at least one other task. The Pareto front is the collection of all systems for which no other system exists that is better at all tasks at once. Shoval et al. showed that when two tasks are important for fitness, species should fall on a line segment in the space of traits. The ends of this line are called archetypes: phenotypes optimal at one of the two tasks. If three tasks are important, data should fall on a plane and within that plane on a triangle. The vertices of the triangle are the three archetypes. Four tasks lead to a tetrahedron and so on. The position of each species in this polygon or polyhedron relates to the relative importance of the tasks in its niche (Fig 1C–1F): specialist in one task should be near the corresponding archetypes, and generalists should be in the middle of the polygon. To apply this theory, one does not need to know the tasks in advance. If the data is found to fall in a polygon with clear vertices, the tasks can be inferred: clues about the tasks are gained by noting the special features of the animals closest to each archetype- these features provide hints about the task optimized by that archetype [37]. The tradeoffs in the Pareto theory can result in either positive or negative correlations between traits (Fig 1G), and thus differ fundamentally from the tradeoffs of Stearn’s life history theory. Pareto theory was previously used to identify tasks in animal morphology [35,38,39], behavior [40] and gene expression [35,37,41,42]. It was not previously applied to life-history traits.

We find that the mass-longevity dataset for mammals and birds is well-described by a triangle in trait space. The triangle is filled nearly uniformly. We demonstrate how the theory can be used to infer three tasks from the mass-longevity triangle. A putative fourth task is identified by analyzing additional life history traits. This provides a new coordinate system for comparative studies of animal longevity, and more generally an approach for understanding the geometry of life history trait variation.

## Results

### Longevity-Mass for mammals and birds falls approximately in a triangle

To comprehensively study the geometry of life history space of endothermic animals (mammals and birds), we analyzed a large dataset of life history traits taken from AnAge build 13 [43]. We filled some of the gaps in the database by literature search, adding 20 longevity values for species with missing data, and 168 values for other life history traits (Table 1 summarizes the taxa, and S1 Table lists the entire data). We also added brain size from a different source [17] resulting in 324 additional entries. We analyzed species with both mass and longevity known, resulting in a dataset with 2105 endotherms, each with 13 relevant life-history features (See Methods and S1 Table).

**Table 1:** Summary of taxa used in this manuscript.

Aves 1086	Mammalia 1019
Accipitriformes 56	Afrosoricida 6
Anseriformes 64	Artiodactyla 153
Apodiformes 18	Carnivora 162
Apterygiformes 1	Cetacea 42
Bucerotiformes 5	Chiroptera 87
Caprimulgiformes 5	Cingulata 8
Casuariiformes 4	Dasyuromorphia 25
Charadriiformes 123	Didelphimorphia 16
Ciconiiformes 14	Diprotodontia 52
Coliiformes 1	Erinaceomorpha 7
Columbiformes 16	Hyracoidea 2
Coraciiformes 13	Lagomorpha 12
Cuculiformes 5	Macroscelidea 5
Eurypygiformes 1	Monotremata 3
Falconiformes 16	Peramelemorphia 9
Galliformes 36	Perissodactyla 16
Gaviiformes 3	Pilosa 5
Gruiformes 17	Primates 152
Musophagiformes 1	Proboscidea 2
Otidiformes 1	Rodentia 230
Passeriformes 397	Scandentia 5
Pelecaniformes 40	Sirenia 3
Phaethontiformes 2	Soricomorpha 16
Phoenicopteriformes 3	Tubulidentata 1
Piciformes 23	
Podicipediformes 5	
Procellariiformes 30	
Psittaciformes 138	
Sphenisciformes 4	
Strigiformes 23	
Struthioniformes 1	
Suliformes 19	
Tinamiformes 1	

**Table 2:** Mass and longevity of selected animals.

Species	Mass (gr)	Longevity (years)
Whales	(3±1)⋅10^7^	80±10
Elephants	(4.0±0.8)⋅10^6^	65.2±0.2
Hippopotamus	4⋅10^6^	61
shrews	4.5±0.9	3.2±0.3
mice	7.3±0.6	3.5±0.2
hummingbirds	3.2±0.1	5±1
bats	7.7±0.7	29±5
myotis	7.2±0.2	21±1
naked mole rat	35	31

The values for each animal are the median and median deviation of the taxa.

**Fig 2:**
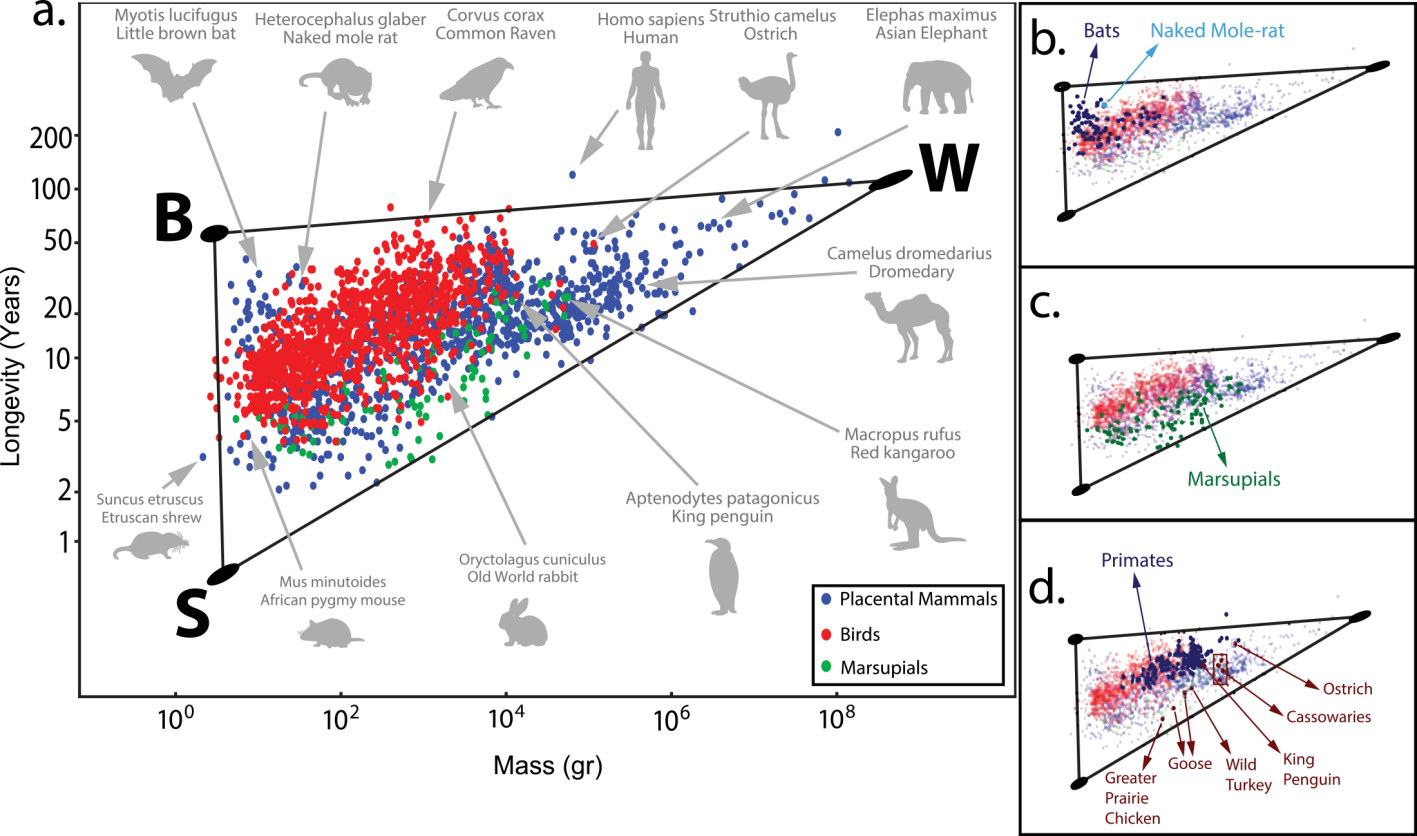
The longevity-mass data set is well described by a triangle in log space. (a) Placental mammals (blue), birds (red) and marsupials (green) are presented in a log-log plot, black lines represent the best fit triangle and the three ellipses on the vertices represent the position and error of the archetypes determined by bootstrapping (see Methods). (b) Bats, naked mole rat are close to the B archetype. (c) Marsupials cover much of the same triangle. (d) Primates are close to the B archetype, flightless birds are far from it.

We begin by focusing on mass and longevity (Fig 2A). We find that the data is much better explained by a triangle (the minimal area triangle enclosing the data up to a defined number of outliers is shown in Fig 2A, see Methods, and S1 Text) than by a line such as an allometric power law (Fig 1B), as well as higher order polygons such as a four-vertex polygon (quadrangle). Statistical model selection tests (Akaike information criterion, cross validation tests) indicate that a triangle is a better fit than a line, even when taking into account that fitting a triangle requires more parameters than a line (see Methods). Fits to higher order polygons (quadrangle, pentagon etc.) do not provide enough improvement in fit quality to justify their extra parameters. We also tested the statistical significance that a triangle describes the data by comparison to randomized data, according to a test developed in [35], (*p* = 0.005, see Methods). We conclude that a triangle is a good description of the geometry of the data in the log mass-log longevity plane.

We also asked whether the way longevity is defined- the longest lived animal on record- may affect the geometry. We aimed to allow for a shorter longevity value for each species, perhaps reflecting natural conditions. We thus generated a dataset in which longevity of each species was multiplied by a random number between 0.8 and 1.0, thus shortening its longevity entry. We find that a triangle still significantly fits the data in over 95% of 1000 such randomly perturbed datasets (See S1 Text).

Two of the edges of the mass-longevity triangle are approximately parallel to the axes. The third edge has a slope of 0.274±0.007. This value agrees with the ¼ allometric relation between mass and longevity (Fig 2A), *L* ∼ *M* ^1/4^. Linear regression applied to the full dataset also gives a slope close to ¼, when ‘outliers’ such as bat species are removed, explaining previous findings of an allometric ¼-law.

The three vertices of the triangle correspond–in Pareto theory- to archetypes. The error in the vertex position is about 3% as estimated by bootstrapping (Fig 2A). The first archetype is close to high mass–high longevity species such as whales, elephants, and the hippopotamus. We name this the W-archetype, for whales. The second archetype is next to species of low mass and low longevity such as shrews, mice and hummingbirds. We name it the S-archetype, for shrews. The third archetype is near low mass—high longevity species. These include bats and the bat-like myotis, the naked mole rat, and birds like canaries. We name this the B-archetype for bats (for values of mass and longevity see Table 2).

Flying birds lie in a region closest to the B (bat) archetype (Fig 2A). Marsupials (in green) are spread over much of the triangle (Fig 2C). Non-flying birds lie farthest from this archetype (Fig 2D). Primates are relatively close to the B archetype (Fig 2D), as are arboreal squirrel species (S1 Fig).

A phylogenetic analysis of this dataset shows that above the taxonomic level of family, the position on the triangle cannot be wholly explained by phylogenetic history (S2 Text and S2 Fig).

### The S-archetype (shrews, rodents) with short lifespan and high predation is enriched with high litter size and litter frequency

We next asked which tasks might be at play in each archetype. We approach this by looking for commonalities in the species near the archetypes. Quantitatively, we ask which life history features (other than mass or longevity) are maximal or minimal at the archetype. If Pareto theory is applicable, certain traits should be maximal or minimal in animals closest to the archetype; these traits should decline (or rise) with distance from the archetype.

The S-archetype lies at low mass and short lifespan. The closest animals are shrews and rodents (orders *Rodentia* and *Soricomorpha*). We tested 11 different life history traits of the animals from the AnAge database [43] as a function of their distance from the S-archetype. We normalized traits with units of time (female maturity, weaning, inter-litter interval) by the organism lifespan, and normalized traits with units of mass (birth weight, weaning weight) by the adult weight, to obtain dimensionless traits. Some information was lacking in the database, because some species had missing trait values (Ranging from 82% for Body temperature to 28% for Litter/Clutch size).

To search for enriched (depleted) features next to the archetypes, we followed the algorithm of Ref [37]. Briefly, we divided species into bins according to their distance from a given archetype, such that each bin had the same number of species. Then, we looked for features that have significantly maximal (minimal) values in the bin next to an archetype. According to Pareto theory, traits associated with the task of the archetype should have their maximal or minimal value at the bin closest to the archetype.

**Fig 3:**
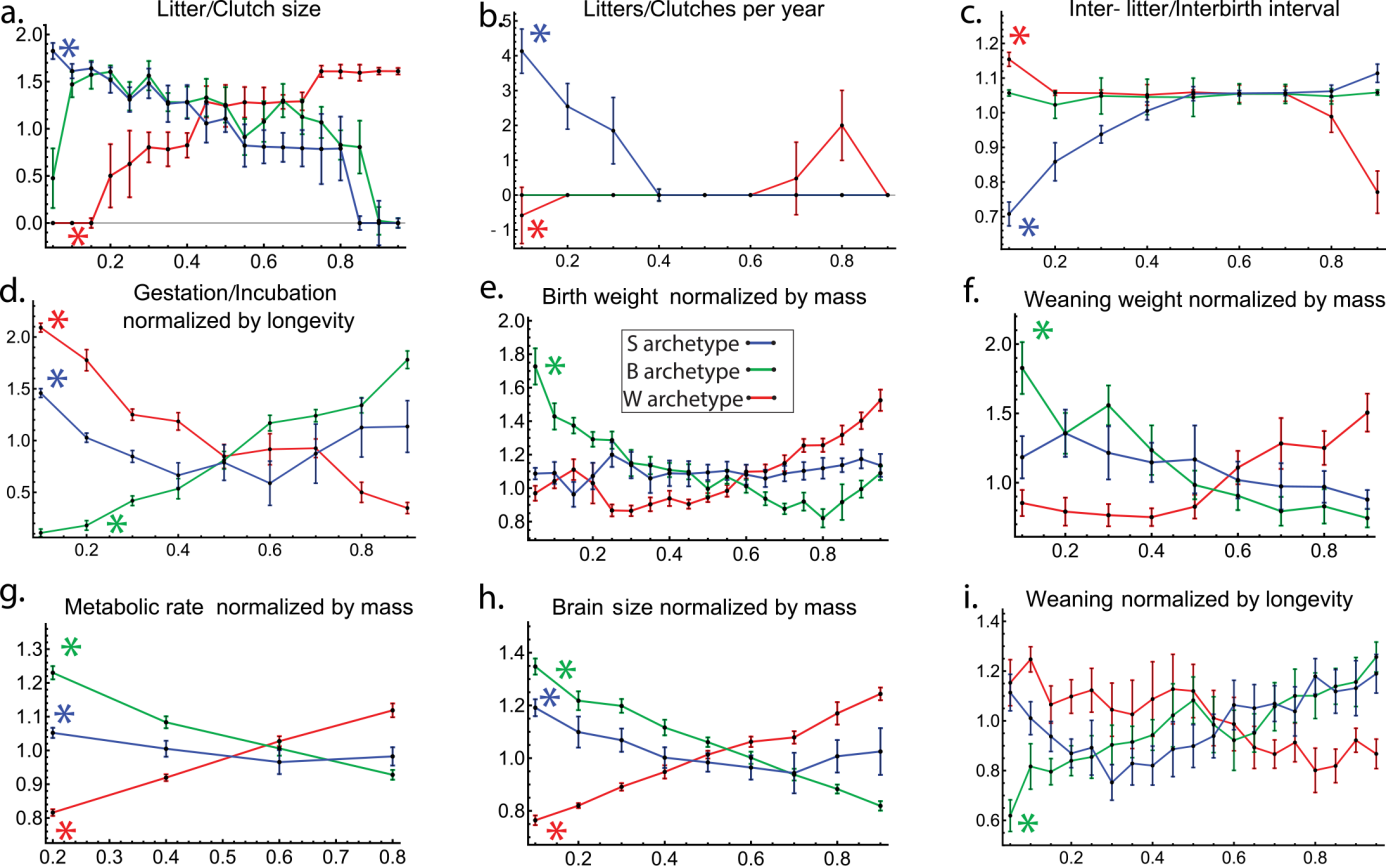
Each archetype is enriched with specific life-history traits. Trait values are plotted versus distance from an archetype. Species were binned to equal sized bins according to distance from an archetype. Median trait value in the bin normalized by median trait value in the dataset is shown, error bars are from bootstrapping. Curves for features enriched significantly at an archetype are marked with an asterisk (listed also in Table 3). Bin size selection is explained in S3 Text.

We find (see Methods, S3 Text, including multiple hypothesis tests) that several life history traits are highly enriched near the S-archetype (Fig 3 and Table 3). Specifically, two traits associated with high reproduction rate—litter/clutch size and litter/clutch per year—are both maximal at this archetype. In addition, normalized brain size (for mammals) is highest at this archetype, as well as the relative gestation/incubation period relative to lifespan (see Fig 3).

**Table 3:** Enrichment analysis of the archetypes.

Archetype	Trait	Median difference	First bin
W	Brain Size normalized by mass	-0.74	Minimal
W	Gestation/Incubation (days) normalized by longevity	0.52	Maximal
W	Inter-litter/Interbirth interval	0.24	Maximal
W	Litter/Clutch size	-0.40	Minimal
W	Litters/Clutches per year	-0.046	Minimal
W	Male maturity (days) normalized by longevity	0.090	Maximal
W	Metabolic rate (W) normalized by mass	-0.55	Minimal
W	Temperature (K)	0.0015	Maximal
B	Birth weight (g) normalized by mass	0.46	Maximal
B	Brain Size normalized by mass	0.52	Maximal
B	Gestation/Incubation (days) normalized by longevity	-0.44	Minimal
B	Male maturity (days) normalized by longevity	-0.094	Minimal
B	Metabolic rate (W) normalized by mass	0.42	Maximal
B	Temperature (K)	-0.0013	Minimal
B	Weaning (days) normalized by longevity	-0.28	Minimal
B	Weaning weight (g) normalized by mass	0.24	Maximal
S	Brain Size normalized by mass	0.31	Maximal
S	Gestation/Incubation (days) normalized by longevity	0.22	Maximal
S	Inter-litter/Interbirth interval	-0.85	Minimal
S	Litter/Clutch size	0.30	Maximal
S	Litters/Clutches per year	0.30	Maximal
S	Metabolic rate (W) normalized by mass	0.099	Maximal

The enrichment for these traits is rather sharply peaked at the bin closest to the archetype, and falls off rapidly with distance from the archetype. This is the behavior expected from Pareto theory. These results support the conclusion that a task including rapid reproduction, many offspring, and rapid growth is maximized at the S-archetype. This inference makes sense: these animals are generally thought to face high predation. High predation has been suggested as an evolutionary pressure for resource allocation to early life, which correlates with short lifespan [1,8,44] in mammals (but see exception [45] in guppies). The tasks most important in this situation may be rapid reproduction, many offspring, and rapid growth [27].

The S-archetype is a point deduced from the overall shape of the dataset- an extrapolated vertex of a triangle that encompasses the dataset; the region in the immediate proximity of the S-archetype is empty. This is because the endotherms in the dataset do not have longevity shorter than 2 years. There may be a physiological or ecological limit that prevents endotherms from going below a minimal longevity. For example, the heart-rate of mammals smaller than a shrew is expected to approach the physiological limitations of the circulatory system [46].

### W-archetype (whales, elephants) with large mass, long-term survival and low predation is enriched with low clutch-size and clutch-frequency

The closest animals to the W-archetype are whales, elephants, hippopotamus and orcas, which have large mass and high longevity. Enrichment analysis shows that at this archetype, animals have relatively long gestation/incubation periods. They have smallest and least frequent clutches/litters [47] (Fig 3). They have a relatively small brain compared with their mass. Again, the enriched traits are maximal at the bins closest to the archetypes, and decay with distance from the archetype.

The task inferred for this archetype is appropriate for these large animals according to previous literature. High mass may allow them to escape predation, so that selection pressure to live longer becomes more meaningful [48–50]. Furthermore, they invest resources in a single offspring, which probably has good odds to reach adulthood.

### B-archetype (bats, naked mole rat) corresponds to a protected niche that provides long term survival and low predation, and is enriched with high relative weaning weight, low relative gestation and incubation

The third archetype, the B-archetype, has a low mass (close to the mass of the S-archetype) but high longevity values (almost half of the longevity of the W-archetype). The animals closest to this archetype are bats, the naked mole rat, and many songbirds such as the Eurasian goldfinch, Broad-tailed hummingbird and Purple sugar bird. These species are thought to have lower extrinsic mortality rates by finding niches that reduce the number of possible predators. Both bats and birds fly, whereas the naked mole rat lives underground.

Somewhat farther from the archetype are arboreal species (primates, Southern flying squirrel, Pygmy marmoset, Sugar glider), which also have relatively low predation due to their niche (Fig 2) [10,51]. Among birds, there appears to be a correlation between flying ability and the distance from this archetype, where the birds farthest from this archetype are flightless, such as the penguin, ostrich and hen (Fig 2D). Related species occur in layers at different distance from this archetype, suggesting different levels of protective environments. The bats are closest to the archetype, then the primates. In a similar distance from the archetype are the family of *Sciuridae* (squirrels), and farther away a strip with a high concentration of “armored” species such as hedgehogs, pangolin, armadillos and porcupines (S1 Fig).

Enrichment analysis indicates that species closest to the B-archetype have shorter pregnancies (or incubations) relative to lifespan. They wean the fastest relative to their longevity. This means that a larger part of their life is spent in puberty and adulthood. Compared to their mass, these species have large brain and they are born large and wean large. (Fig 3) These traits are maximally enriched at the archetype and decay rapidly with distance from it.

### Evidence for a fourth archetype–large predators

We went beyond two-dimensional trait-space of mass and longevity, by considering additional life history traits. One challenge is missing data- many of the 13 traits in the database are sparsely filled. We therefore chose the four traits with the most data, in the sense that there is the largest number of species in the database with all four traits available. These traits are mass (adult weight), longevity, female maturity and birth weight (see Methods). There are 550 mammals with data in all four traits. We removed 142 marsupials from the dataset, because birth weight in marsupials has a different physiological context and clusters far from other mammals.

We analyzed the species in this four dimensional trait space. We performed principal component analysis (PCA) to determine the effective dimensionality of the data. We find that the first 3 PCs capture ~99.5% of the variance (p < 10^−4^ compared to shuffled data).This suggests that data falls effectively in three dimensions. The three PCs are loaded heavily with the following traits: PC1 is related to mass (adult weight and birth weight), PC2 to time (longevity and female maturity), and PC3 to the ratio between the adult weight and birth weight.

**Fig 4:**
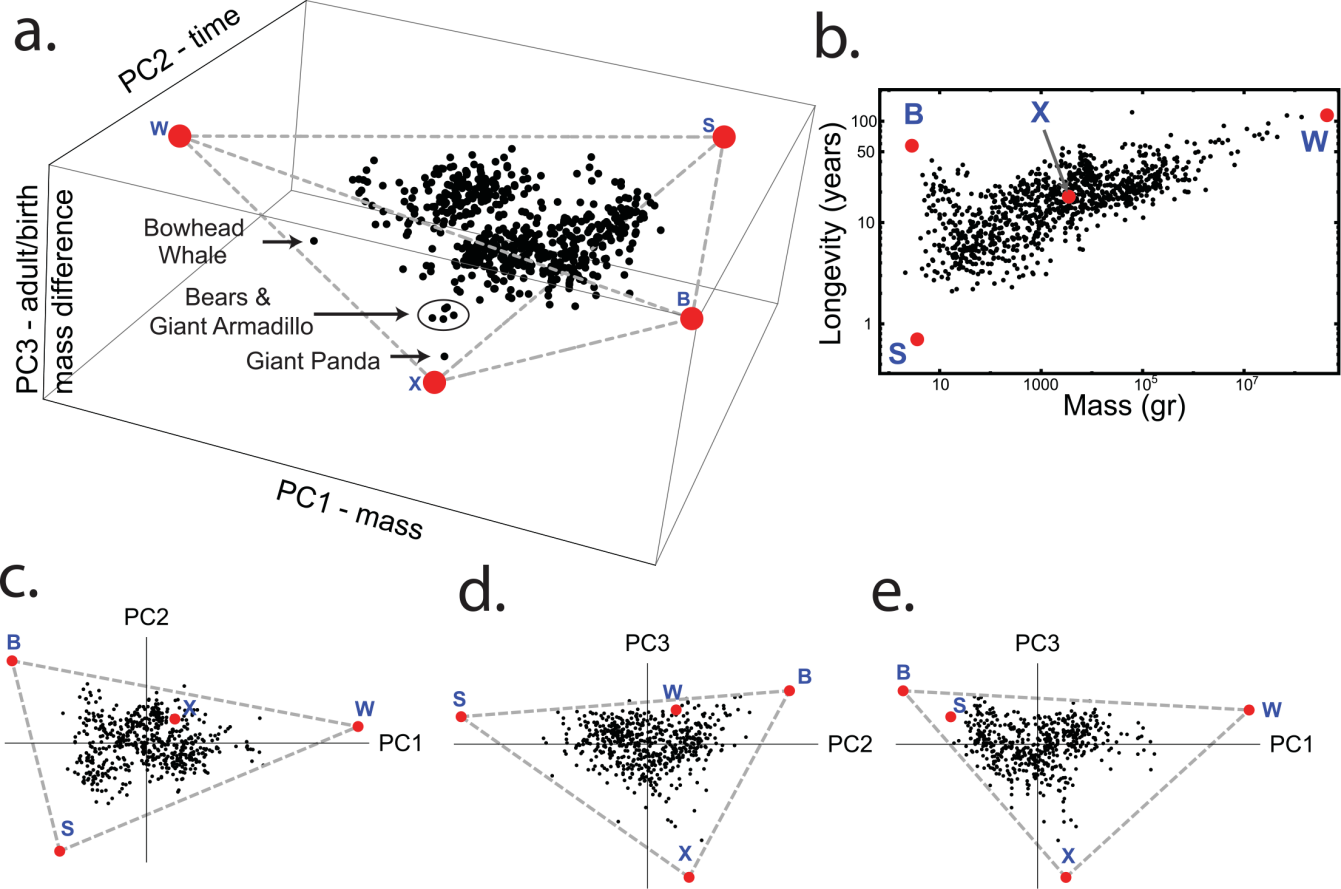
Mammalian species fall approximately in a tetrahedron when considering a higher dimensional space of life-history traits. (a) Axes are linear combination (PCs) of: adult weight, birth weight, longevity, and female maturity. PC1 is primarily related to mass (adult weight and birth weight), PC2 to time (longevity and female maturity), and PC3 to the difference between the adult weight and birth weight. The archetypes B, S, and W are indicated, and the new fourth archetype is marked by X. Species closest to the fourth archetype are indicated. The best fit tetrahedron is indicated in grey dashed lines; vertices (archetypes) are marked in red. (b) The new archetype (X) falls near the middle of the mass-longevity triangle. (c-e) The projections of the data and tetrahedron on the three principal planes are shown for additional perspective.

We went on to test whether the data can be reasonably described by a polyhedron in this three dimensional space (Fig 4A). We find that a tetrahedron captures the data well, and with high significance compared to randomized data (p < 2⋅10^−4^) [37] (see Methods). Higher-order polyhedra do not improve the fit to justify their extra parameters (Methods).

Fig 4C–4E shows this tetrahedron from three points of view in the space of the first 3 principal components. The tetrahedron has four vertices, which we consider as four archetypes. Three of the archetypes match those we discussed so far. The fourth archetype lies at an intermediate point of mass-longevity (M~3.5kg, L~18 years) (Fig 4B), and at low birth weight. The animals nearest this archetype are the panda and bears, while somewhat farther are other carnivorous mammals (order *Carnivora* including the feline family—lions and tigers, and the canine family wolves). Enrichment analysis did not supply significant enrichment of other life-history traits at this archetype, perhaps due to the small number of data points. The potential task or strategy related to this archetype is thus mainly performed by large predators.

### Pareto theory offers an explanation of triangles in trait space

We conclude by discussing previous work in the context of Pareto theory. In the field of life history, several triangle-shaped suites of variation were found in the past (summarized in S2 Table). Grime [52] considered a trait space of plants, including life history traits such as leaf longevity and litter, and morphological traits such as root morphology and leaf from. This study identified three syndromes of traits that go together. Each syndrome is specialized for a different strategy: stress tolerant (S), ruderal (R), and competitive (C), which apply to extreme niches: High stress and low disturbance, low stress and high disturbance, and low stress and low disturbance. The C, S and R survival strategies can be considered as tasks. The CSR model is widely used when describing plants life history strategies, and was implemented also to reef corals [53] and fungi [54]. The original CSR model by Grime was depicted in performance space, by triangle whose vertices represent each strategy. More recent work [53] analyzed trait space using principal coordinate analysis (PcoA), to find that high dimensional trait data falls approximately on two dimensions. Projecting on this 2D plane, data falls in a triangle, with generalists in the middle and specialist at each strategy towards the vertices. This series of studies fits well with Pareto theory, where the vertices of the triangles in trait space define three archetypes, whose traits correspond to Grimes syndromes.

In another pioneering study [55] a triangle was found in the life history trait space of fresh and marine fish. Here the triangle was found in trait-space defined by the measured juvenile survival, fecundity, and onset and duration of reproductive life. The three strategies (tasks) matching three niches in this case are: (i) periodic strategy in environments with predictable patterns of change, (ii) opportunistic strategy in marginal habitats and large predation, and (iii) equilibrium strategy in temporally stable and resource limited environments. Evidence for a triangle due to different resource limitations was found in phytoplankton, where competitive ability for nitrate, phosphate as well as cell volume exert tradeoffs [56]. In many of these studies it is noted that the strategies described are extremes, while intermediate strategies lie in the middle of the triangle. These studies thus seem to be consistent with predictions of the Pareto theory of Shoval et al.

## Discussion

The present study indicates that variation in longevity and other life history traits can be understood using the multi-objective optimality approach of Shoval et al [35]. We studied life history traits in a large dataset of endothermic animals. We find that mass and longevity fall in a triangle, and not a line, suggesting three major life history strategies. Analysis of additional traits suggests a tetrahedral geometry with a fourth putative strategy related to large carnivores. Using the multi-objective optimality approach offers a new theoretical framework and a testable model for future research. It also offers a new way to consider tradeoffs that complements Stearns theory [34].

This study demonstrates how Pareto theory can be used to systematically infer tasks from the dataset geometry. The triangular geometry of longevity-mass suggests three tasks or strategies. Animals closest to the vertices are predicted to specialize in particular strategies, and to have extreme values of certain life history traits (other than mass and longevity). This prediction is fulfilled. These enriched features provide clues to the possible tasks at play. The three putative strategies of whales, shrews and bats are summarized in Table 3. They relates to classical notions of fast and slow strategies (fast reproducing shrew versus long-lived whale), with a third strategy for animals that are thought to have low predation niches (bats, arboreal animals, flying birds etc.). Animals in the middle of the triangle are generalists; their distance from the different vertices suggests their relative use of the different strategies. The three-strategy picture that emerges for endothermic animals echoes the three-strategy picture for plants (competitive, stress tolerant and ruderal) [52] and fish [55]. A fourth strategy, related to large carnivores, remains to be better defined when more data becomes available, and has the potential to further enhance our understanding of life histories interplays and tradeoffs.

This study also makes a distinction between different definitions of the concept of trade-offs (Fig 1G). In life-history theory [34], tradeoffs are defined by negative correlations between measured features such as survival and fecundity. In terms of Pareto theory, features such as survival and fecundity are understood as performances in tasks. Stearns theory thus treats performance space as opposed to *trait* space. Correlations in trait space result from the fact that no phenotype can be optimal at multiple tasks. This leads to *negative or positive* correlations in trait space (traits such as mass, longevity, litter size), and also to the possibility of triangles and other polygons/polyhedra.

It is of major interest to identify genetic and molecular changes that cause differences in longevity. To date, most studies use model species such as *C*. *elegans* [57] and mice [58–60]. Using the present triangle and future molecular data on a very large number of mammalian species [3,61,62], one can hope to gain sufficient statistical power to address longevity in a new way. In particular, using many species may help discard molecular changes that are irrelevant, and to highlight the changes that directly relate to mass-longevity. For this purpose, one can correlate molecular changes with the distance of each species from the three archetypes.

In summary, biological life-history traits, traditionally analyzed using allometric lines and multidimensional linear regression, can be shown to have more complex configurations such as triangles, tetrahedrons and maybe even higher dimensional simplexes. Pareto theory provides a theoretical framework to understand such polygons. The vertices of these shapes correspond to specialists at key tasks that combine to generate fitness in different niches, and the edges are the tradeoffs between such two tasks. Mass and longevity of mammals and birds fall on a triangle and not an allometric line, suggesting three main tasks or strategies. These strategies were analyzed using a systematic way to infer life-history tasks- by noting which features are enriched near the archetypes. A novel fourth task/strategy related to carnivores is suggested at the next level of resolution of the Pareto approach. Future work can employ Pareto archetype analysis to other life-history traits with the hope of better understanding biological diversity in form and function.

## Methods

### Life-history data

Data was downloaded from AnAge database of animal longevity, build 13 [43]. We considered 13 traits: female maturity (days), male maturity (days), gestation/incubation (days), weaning (days), litter/clutch size, litters/clutches per year, inter-litter/interbirth interval, birth weight (g), weaning weight (g), adult weight (g), maximum longevity (yrs), metabolic rate (W), and temperature (K). Maximum longevity is manually curated data on the oldest age on record [63]. Values of other traits were averaged across measurement methods, geographical location and literature source [63], with field data preferred over captive data. When there was a substantial difference between age at sexual maturity and age of first reproduction, the former was used. Sexual maturity is defined as the time from conception to physiological sexual maturity [9]. Further information about AnAge database can be found in refs [9,43,63] and on the website: http://genomics.senescence.info/help.html#anage.

Four traits in the AnAge database were not used: growth rate because it is a fitting parameter of a model where for birds and mammals different models were used, body mass because it strongly and tightly correlates with the adult weight entry; we also did not use mortality rate doubling time (MRDT) for a given species using the Gompertz equation and the infant mortality rate (IMR). Because of missing data, we added to this dataset information from several sources on longevity and other life history traits [64–67]. We consider only species with data in both adult weight and longevity (S1 Table lists the data), which amounts to 2105 endotherms (rows), each with its taxonomic tree and 13 relevant life-history features (columns). See Methods for more details. We also added the brain size from [17]. We took only the species from this database that existed on AnAge this resulted in 324 values for brain size.

### Fit to polygons and model selection

We tested several models for the log mass-log longevity data. This includes a linear fit, *l* = *am* + *b*, a triangle enclosing he data and more generally an *n*-vertex polygon. We first used the PCHA algorithm to find the best *n* points on the convex hull of the data that accounts for most of the variance in the data as described in [41]. A line is defined by two archetypes, a triangle by three etc. For each number of archetypes *n*, we compute the explained variance given by the mean relative distance of the *N* data points to the polygon $EV(n)=\frac{1}{N}\sum_{i=1}^{N}\left(1-\left\|p_{i}-s_{i}\right\|/\left\|p_{i}\right\|\right)$. Here *p*_*i*_ is the *i*th data point and *s*_*i*_ is the closest point to *p*_*i*_ in the polygon [41,68]; points inside the polygon have ‖*p*_*i*_ − *s*_*i*_‖ = 0. The normalization term ‖*p*_*i*_‖, refers to the distance to the center of mass of all the data points. We seek a number of archetypes for which adding an additional archetype does not increase *EV* by much. We find that explained variance for n = 2, 3, 4, 5 is EV = 0.9711, 0.9989, 0.9993, 0.9999. Thus a triangle is better than a line, and four-vertex or five-vertex polygons make only a tiny improvement over the triangle (2% vs. 0.04%).

We approached model selection also in a second way, by using a criterion that takes into account the number of free parameters in the model, namely the Akaike information criterion (AIC)[69]. The first step in calculating AIC is to calculate how likely each model is. Likelihood is calculated by assuming that the data was drawn from a distribution (the model). According to standard practice, we used a Gaussian error model: the convolution of the line/triangle/polygon with a Gaussian to account for the possibility of noise. The standard deviation of the Gaussian distribution was chosen to be 30% of the value at the data-point. Similar results were obtained with a 10% or 100% std. In all of these cases the triangle was overwhelmingly more likely than a line (AIC/2 ~–log likelihood ~ 7500, 3600 for line and triangle in the 30% case, 56000, 5600 for the 10% case, and 4300, 3900 in the 100% case). A triangle was more likely than a 4-vertex model for the 30% and 100% cases.

### Statistical significance of triangle

To estimate the statistical significance of the description of the data by a triangle or a tetrahedron (polygon), we calculated the t-ratio. The t-ratio is the ratio of the polygon’s volume to the volume of the convex hull of the data [35]. It is a measure for the extent that the data fills the polygon. A t-ratio of one occurs when the data convex hull is exactly the desired polygon. We then generate randomized datasets by sampling from the cumulative distribution created by each coordinate independently from its ensemble of measured values. This eliminates correlations between traits while conserving the distribution of values of each parameter. We calculate the t-ratios for each randomized dataset in comparison to its own minimal volume enclosing polygon, and set the p-value to be the proportion of randomized sets with a smaller or equal t-ratio than the original data. See S1 Text for more details.

### Archetype position and error

In order to determine the position of the three archetypes on the mass-longevity plane we used the algorithm described by Shoval et al. [35]. This algorithm finds the minimal triangle enclosing all the data point, without allowing any outliers. To be robust to outliers, we applied a peeling procedure–removing the convex hull of the data. We repeated this procedure, and after each peeling we looked for the archetypes of the new dataset. The change of position of the W archetype was large after one peeling step (~17% of the large edge of the triangle), but remained relatively unchanged after more peelings (~3%). Thus, the final position of the archetypes presented here is given after the data was peeled once.

All the data points inside the polygon obey the rule, *X* = *θA*. Here, *X* is the matrix of the data (namely, mass and longevity), *A* is the matrix of the archetypes, where each column is a different archetype and *θ* is the weight vector representing the compromises between the tasks (archetypes). For each data point ∑*θ*_*i*_ = 1. For every point inside the triangle ∀*θ*_*i*_ ≥ 0. Values of theta are given in S1 Table.

We computed the uncertainty of the archetype positions by bootstrapping, resampling the 2105 data point with replacements and computing the new triangle archetypes. We obtained archetype position distribution by repeating this procedure 1000 times. The standard deviations of the archetype positions are depicted as ellipses in Fig 2.

### Phylogeny and position on the triangle

The intersection between the data in the phylogeny of [70] and the present dataset includes 966 mammals. We represented each data points by its first two weights (the third is determined by the demand that ∑*θ*_*i*_ = 1). On this resulting triangle we measured distance of two species, *d*, by the Euclidean distance between the points. The distance on the phylogenetic tree was measured as the shortest path distance, namely, for each two species, we looked for their nearest common ancestor, and summed the length (years) of the branches leading to each species (see S2 Text and S2 Fig).

### Enrichment

For each archetype, we divide the data into equally populated bins according to their distance from the archetype. Bin size was determined using a bootstrapping test (see S3 Text). Then we check whether the median of each trait in each bin is maximal (or minimal) with respect to the rest of the bins. We then test whether the distribution of points in the first bin is significantly different than the rest of the data (the Mann-Whitney test [71]). Then we used the Benjamini-Hochberg procedure for multiple hypothesis testing [72]. All the enriched features in Table 3 are significant after the multiple hypothesis testing. For further explanations on the way we tested for feature robustness and the choosing of bin-size see S3 Text.

### Higher dimensional trait space and the fit tetrahedron

We scanned all 715 possible combinations of four traits of the 13 in the database, and chose the four traits with the largest number of species that have data on all four traits. We found the best fit tetrahedron using SISAL algorithm [73], evaluated the errors in archetypes by bootstrapping (12% errors), and the significance by the t-ratio test (*p* < 2⋅10^−4^).

## Supporting Information

S1 Fig Taxa with protected niches lie close to the B archetype. The different species are plotted in the mass-longevity plane (gray), where the *Chiroptera* order (bats) is in green, the order *primates* in black, the family *Sciuridae* (squirrels) in blue, and the orders *Erinaceomorpha* (hedgehogs), *Cingulata* (armadillos) and the families *Hystricidae* (Old World porcupines) and *Erethizontidae* (New World porcupines) in red.
(PDF)

S2 Fig Phylogenetic history only partially explains position on the triangle. (a) Distribution of species at different taxonomic levels. Ellipses represent the median deviation around the median at each level: Classes, orders, families and genera. **(**b) Phylogenetic tree, color coded according to position on the mass-longevity triangle. Blue, red and yellow indicate closeness to the S, W, and B archetypes respectively (see inset). Highlighted are cases of related species with very different positions on the triangle. c. Phylogenetic distance (in log millions of years) versus distance on the mass-longevity triangle. The four crosses represent the median and median deviation for each taxonomic level.
(PDF)

S1 Text Statistical significance of triangle. The database we used includes maximum longevity values. Maximum longevity can be viewed as extreme values of a distribution of the actual life span of species, and longevity in the field may be lower than in captivity.
(DOCX)

S2 Text Phylogeny only partly explains position on triangle. The position of a species within the triangle is determined by phylogenic history together with selective pressures in its niche.
(DOCX)

S3 Text The enrichment sensitivity to bin-size. We choose the bin-size for the enrichment analysis so that the enriched features remain robust to outliers and noise, and remain enriched in a broad range of bin-sizes.
(DOCX)

S1 Table The list of all the analyzed species. The dataset we used [43], the first 6 columns represent the taxonomy and name of the species. The next 13 columns depicts the 13 life history traits we used in our analysis, the next 3 columns show the weights of each archetype for each data-point on the mass-longevity triangle, and the last 4 columns show the weights of each archetype for each data-point on the tetrahedron.
(XLS)

S2 Table Previous studies found triangles to describe organism distributions. The first 3 columns show the journal reference of previous studies in which triangles were used to describe the data. The last 4 columns describe how the paper relates to the data, as tasks, traits or characteristic.
(DOCX)

S3 Table Closely species can lie far on the mass longevity triangle. Here we show the taxonomy of closely related species and their mass and longevity values.
(DOCX)

S4 Table Far species on the phylogenetic tree can converge to close values on the mass longevity triangle. Here we show the taxonomy of species and their mass and longevity values.
(DOCX)
