# Supplementary figures and images for: The Mass-Longevity Triangle: Pareto Optimality and the Geometry of Life-History Trait Space
Source: PLoS Comput Biol. 2015 Oct 14;11(10):e1004524. doi: 10.1371/journal.pcbi.1004524 (PMC4605829; doi:10.1371/journal.pcbi.1004524)

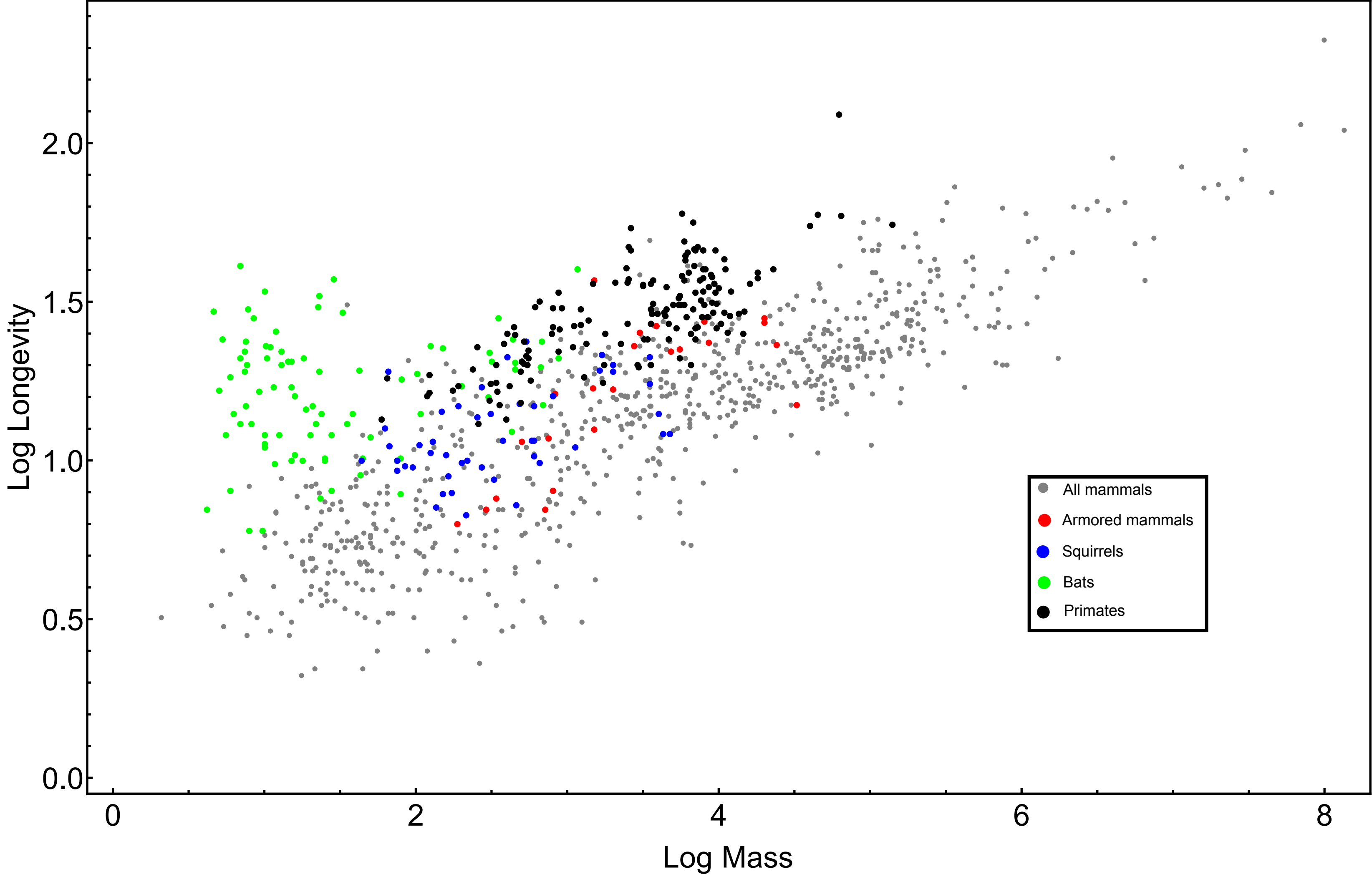

Supplement: S1 Fig — The different species are plotted in the mass-longevity plane (gray), where the Chiroptera order (bats) is in green, the order primates in black, the family Sciuridae (squirrels) in blue, and the orders Erinaceomorpha (hedgehogs), Cingulata (armadillos) and the families Hystricidae (Old World porcupines) and Erethizontidae (New World porcupines) in red. (PDF) [file pcbi.1004524.s001.pdf]
