# Supplementary material for: The Mass-Longevity Triangle: Pareto Optimality and the Geometry of Life-History Trait Space
Source: PLoS Comput Biol. 2015 Oct 14;11(10):e1004524. doi: 10.1371/journal.pcbi.1004524 (PMC4605829; doi:10.1371/journal.pcbi.1004524)
Supplement: S2 Fig — (a) Distribution of species at different taxonomic levels. Ellipses represent the median deviation around the median at each level: Classes, orders, families and genera. (b) Phylogenetic tree, color coded according to position on the mass-longevity triangle. Blue, red and yellow indicate closeness to the S, W, and B archetypes respectively (see inset). Highlighted are cases of related species with very different positions on the triangle. c. Phylogenetic distance (in log millions of years) versus distance on the mass-longevity triangle. The four crosses represent the median and median deviation for each taxonomic level. (PDF) [file pcbi.1004524.s002.pdf]

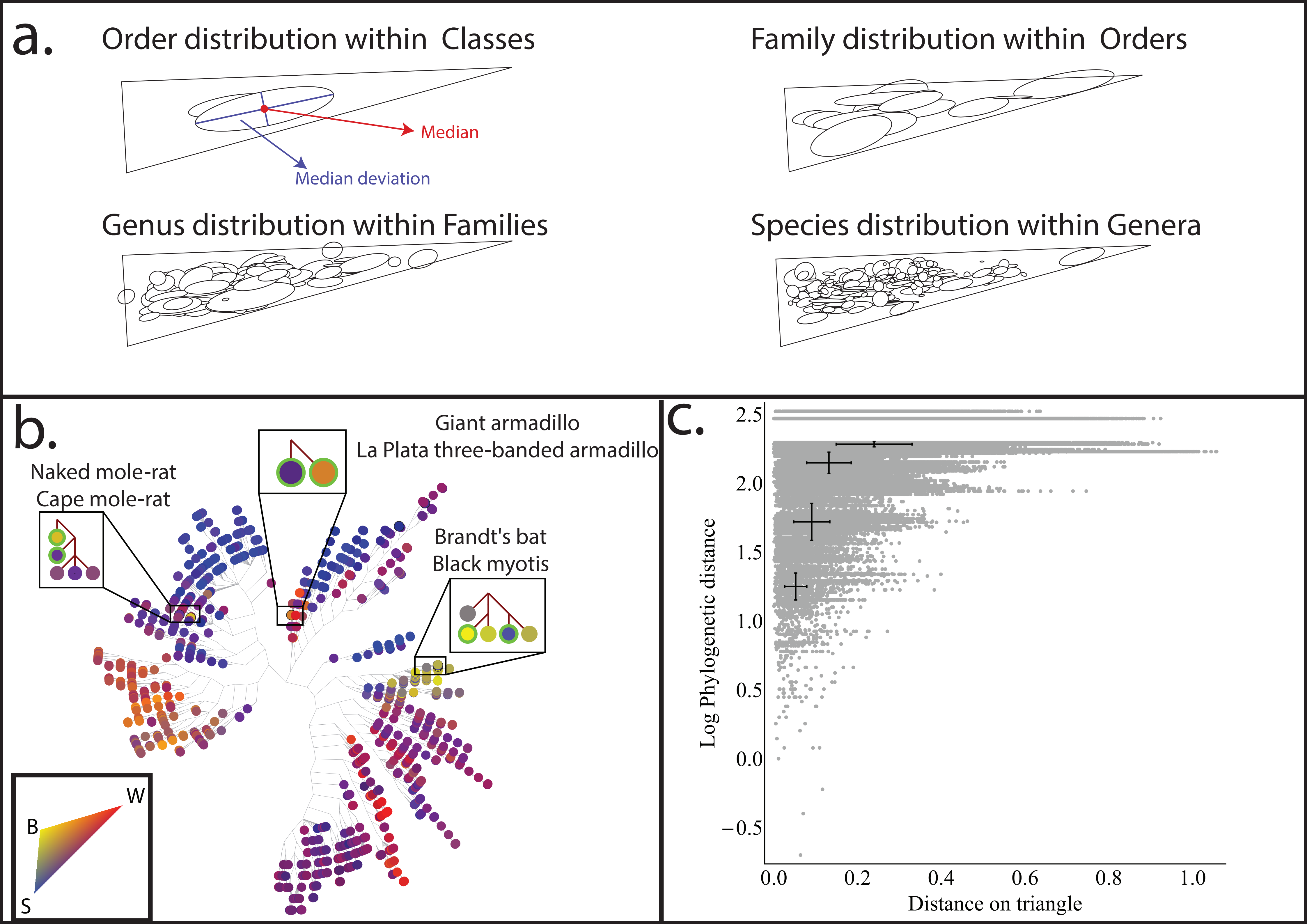
a.
Order distribution within Classes
Median
Median deviation
Family distribution within Orders
Genus distribution within Families
Species distribution within Genera
b.
Naked mole-rat
Cape mole-rat
Giant armadillo
La Plata three-banded armadillo
Brandt's bat
Black myotis
W
B
S
c.
2.5
2.0
1.5
1.0
0.5
0.0
−0.5
Log Phylogenetic distance
0.0
0.2
0.4
0.6
0.8
1.0
Distance on triangle
