# Supplementary material for: The Mass-Longevity Triangle: Pareto Optimality and the Geometry of Life-History Trait Space
Source: PLoS Comput Biol. 2015 Oct 14;11(10):e1004524. doi: 10.1371/journal.pcbi.1004524 (PMC4605829; doi:10.1371/journal.pcbi.1004524)
Supplement: S1 Text — The database we used includes maximum longevity values. Maximum longevity can be viewed as extreme values of a distribution of the actual life span of species, and longevity in the field may be lower than in captivity. (DOCX) [file pcbi.1004524.s003.docx]

Supplementary information 1, for “The mass-longevity triangle: Pareto optimality and the geometry of life history trait space”

Pablo Szekely (1), Yael Korem (1), Uri Moran (2), Avi Mayo (1), Uri Alon (1)

Dept. molecular Cell biology (1) and plant science (2), The Weizmann Institute of Science, Rehovot Israel 76100

**Statistical significance of triangle**

To estimate the statistical significance of the description of the data by a triangle, we calculated the t-ratio. The t-ratio is the ratio of the polygon’s volume to the volume of the convex hull of the data. It is a measure for the extent that the data fills the polygon. A t-ratio of one occurs when the data convex hull is exactly the desired polygon. We generated randomized datasets of the same size as the original data by independently sampling from the cumulative distribution created for each coordinate from its ensemble of measured values. This eliminates correlations between traits while conserving the distribution of values of each parameter. We calculate the t-ratios for each randomized dataset in comparison to its own minimal volume enclosing polygon, and set the p-value to be the proportion of randomized sets with a smaller or equal t-ratio than the original data.

The database we used includes maximum longevity values. Maximum longevity can be viewed as extreme values of a distribution of the actual life span of species, and longevity in the field may be lower than in captivity. We addressed this issue by using maturity instead of longevity and calculating the t-ratio and p-value of the mass vs. maturity data. The maturity data was estimated using the values of female and male maturity. When only one of those values existed we took it, if both of existed we took their minimum. We used the maturity data as an estimate for a lower limit of the lifespan distribution. Here, we find a t-ratio of 0.83 with a p-value smaller than${10}^{-3}$, with similar archetypes.

As another approach to control for possible differences between wild and measured longevity, we created randomized datasets with lower longevity values. We multiplied longevity values by a uniformly distributed random number between 0.8 and 1. We calculated the p-value for fitting a triangle into the new noised data, as explained in Methods. We then repeated this procedure 1000 times. This estimation yielded that ~0.97 of the resulting triangles were significant. The archetypes found were similar to the un-perturbed dataset.

We repeated this with other ranges for the random number that multiplies longevity (between b and 1.0, where above b=0.8). The following table shows the p-value for each values of b.

| Lower boundary for error , b | 0.5 | 0.55 | 0.6 | 0.65 | 0.7 | 0.75 | 0.8 | 0.85 | 0.9 | 0.95 |
| --- | --- | --- | --- | --- | --- | --- | --- | --- | --- | --- |
| Fraction of insignificant triangles | 0.33 | 0.28 | 0.24 | 0.16 | 0.112 | 0.075 | 0.035 | 0 | 0 | 0 |
