# Supplementary material for: The Mass-Longevity Triangle: Pareto Optimality and the Geometry of Life-History Trait Space
Source: PLoS Comput Biol. 2015 Oct 14;11(10):e1004524. doi: 10.1371/journal.pcbi.1004524 (PMC4605829; doi:10.1371/journal.pcbi.1004524)
Supplement: S2 Text — The position of a species within the triangle is determined by phylogenic history together with selective pressures in its niche. (DOCX) [file pcbi.1004524.s004.docx]

Supplementary information 2, for “The mass-longevity triangle: Pareto optimality and the geometry of life history trait space”

Pablo Szekely (1), Yael Korem (1), Uri Moran (2), Avi Mayo (1), Uri Alon (1)

Dept. molecular Cell biology (1) and plant science (2), The Weizmann Institute of Science, Rehovot Israel 76100

**Phylogeny only partly explains position on triangle**

The position of a species within the mass-longevity triangle is determined by phylogenic history together with selective pressures in its niche. To estimate the contribution of phylogenetic history, we compared the position of different species in the triangle to their relationship on the phylogenetic tree. For this purpose, we used the subset of the data where phylogenic information was available (966 out of 1327 mammals), using the phylogeny of Polly et al (Polly 2012). Each species is classified according to its Phylum (*Chordata*), then class (*Mammalia*), order (e.g. *Carnivora*, *Primates*), Family (e.g. *Felidae*, *Hominidae*), Genus and species (e.g. *Panthera* *leo*, *Homo* *Sapiens*).

We evaluated the position of each species on the triangle by its relative distance from each archetype. This yields three dimensionless positive weights which sum to one. The distance between two species is given by the Euclidean distance of their two weight vectors (see Methods). This distance measure, d, ranges between 0 and$\surd2$. We find that species within the same genera tend to lie close on the triangle (average d~0.07), much closer than randomized datasets (which average$d_{rand}\sim0.22$,$p\ll10^{-4}$). Species of the same family but not from the same genus are slightly more spread out on average (d~0.11) but still much less than by chance ($p\ll10^{-4}$), as are species of the same order but not from the same family (d~0.14, $p\ll10^{-4}$). However, at the level of orders (e.g. *Rodentia*, P*rimates*, *Cetacea*) , the species are quite spread out (d~0.26), with median distances that are close to that expected from points that randomly fill out the triangle (p~0.22). We conclude that phylogenetic history is correlated with position on the triangle up to the level of family. At the level of orders and above, that is species separated by more than 190My (million years) on average, the mass-longevity values of species range across the triangle with less correlation to history (Fig S2).

In addition to this broad statistical assessment, we note special cases in which close relatives are far on the triangle (Table S3). Examples include the naked mole rat and the cape mole rat, which belong to the same family (*Bathyergidae*), and are separated by only 63My. Both animals weigh less than 200g, but the cape mole rat lives 11 years and the naked mole rate lives for over 30 years. Thus one lies near the shrew and the other near the bat archetypes. Additional examples of closely related mammals but distant in life history include (i) Brandt’s bat ($m= 7 gr l= 41y$ ) and the Black myotis ($m= 4.2 gr l= 7y$) (same Genus, *Myotis*). (ii) Giant armadillo ($m= 33000 gr l= 15y$ ), and the La Plata three-banded armadillo ($m= 1500 gr l= 36.8y$ ) (same Family, *Dasypodidae*), and (iii) the Black wallaroo ($m= 17500 gr l= 11.8y$ ) and the Northern nail-tailed wallaby (same Family, *Macropodidae*) ($m= 5800 gr l= 5.5y$ ) (Fig 4b). This suggests that the phylogenetic relation does not exclusively determine position on the triangle (Bonsall and Mangel 2004). Similarly, unrelated species can converge to very similar life history traits (Table S4).

**References**

Bonsall, Michael B., and Marc Mangel. 2004. “Life-History Trade-Offs and Ecological Dynamics in the Evolution of Longevity.” *Proceedings. Biological Sciences / The Royal Society* 271 (1544): 1143–50. doi:10.1098/rspb.2004.2722.

Polly, P. David. 2012. “Phylogenetics for Mathematica (Ver. 2.1),” July. https://scholarworks.iu.edu/dspace/handle/2022/14614.
