# Supplementary material for: The Mass-Longevity Triangle: Pareto Optimality and the Geometry of Life-History Trait Space
Source: PLoS Comput Biol. 2015 Oct 14;11(10):e1004524. doi: 10.1371/journal.pcbi.1004524 (PMC4605829; doi:10.1371/journal.pcbi.1004524)
Supplement: S3 Text — We choose the bin-size for the enrichment analysis so that the enriched features remain robust to outliers and noise, and remain enriched in a broad range of bin-sizes. (DOCX) [file pcbi.1004524.s005.docx]

Supplementary information 3, for “The mass-longevity triangle: Pareto optimality and the geometry of life history trait space”

Pablo Szekely (1), Yael Korem (1), Uri Moran (2), Avi Mayo (1), Uri Alon (1)

Dept. molecular Cell biology (1) and plant science (2), The Weizmann Institute of Science, Rehovot Israel 76100

**The enrichment sensitivity to bin-size**

For each archetype, we divided the data into equally populated bins according to their distance from the archetype. Then we asked whether the median of each trait in each bin is maximal (or minimal) with respect to the rest of the bins. We also tested whether the distribution of points in the first bin is significantly different than the rest of the data (using the ranked-sum test (Mann and Whitney 1947)). For each feature we end up with 3 numbers: the difference between the median of the first bin and the median of the entire data, p-value, and whether the first bin is maximal, minimal or neither.

In order to check enrichment robustness to outliers we performed a bootstrapping test to generate 1000 resampled datasets (each time generating N data points by sampling with returns, where N is the original size of the data). For each of these random datasets we performed the same test as described in the previous paragraph.

A feature was considered significant if for at least 90% of the resampled datasets remained significant (p-value), and if in at least 90% of the resampled datasets the first bin remained maximal or minimal.

We repeated this test for each archetype and for each feature with bin sizes: 0.005, 0.01, 0.05, 0.1, 0.2, and 0.5. Many features found robust to outliers stayed enriched in a broad range of bin sizes (~80% of features were found enriched at least 2 bin sizes).

For each feature there is a problem of missing data, thus yielding a different number of points with which calculate enrichment. We required that for each feature in each bin there would be at least 30 points. This demand gave us a lower bound for bin size for each feature: for Litter/Clutch size, Litters/Clutches per year, Birth weight, Female and male maturity, Gestation/Incubation, and Weaning we got a lower bound of 0.05, while for Inter-litter/Interbirth interval, Temperature, Weaning weight, Metabolic rate, and Brain Size we obtain a lower bound of 0.1. We used the bin size 0.2 as an upper bound for all features. For each feature, we used the smallest bin size that has the most enriched and depleted archetypes. The final selection of bin-sizes and features can be found in Table 3.

Mann, H. B., and D. R. Whitney. 1947. “On a Test of Whether One of Two Random Variables Is Stochastically Larger than the Other.” *The Annals of Mathematical Statistics* 18 (1): 50–60. doi:10.1214/aoms/1177730491.

In the table below we show all the significant enrichment of features in various bin sizes, the colored rows correspond to the values we used in the main text.

| Bin Size | Archetype | Feature | Median difference | P-value (Mann Whitney) | First bin is Maximal or Minimal |
| --- | --- | --- | --- | --- | --- |
| 0.05 | B | Birth weight normalized by mass | 0.45662186 | <10^-3^ | Maximal |
| 0.1 | B | Birth weight normalized by mass | 0.372385904 | <10^-3^ | Maximal |
| 0.2 | B | Birth weight normalized by mass | 0.285790029 | <10^-3^ | Maximal |
| 0.5 | B | Birth weight normalized by mass | 0.151455584 | <10^-3^ | Maximal |
| 0.5 | W | Birth weight normalized by mass | -0.207261751 | <10^-3^ | Maximal |
| 0.05 | W | Brain Size normalized by mass | -0.926571083 | <10^-3^ | Minimal |
| 0.1 | B | Brain Size normalized by mass | 0.523965841 | <10^-3^ | Maximal |
| 0.1 | S | Brain Size normalized by mass | 0.314906097 | <10^-3^ | Maximal |
| 0.1 | W | Brain Size normalized by mass | -0.737989143 | <10^-3^ | Minimal |
| 0.2 | B | Brain Size normalized by mass | 0.442651303 | <10^-3^ | Maximal |
| 0.2 | S | Brain Size normalized by mass | 0.275195376 | <10^-3^ | Maximal |
| 0.2 | W | Brain Size normalized by mass | -0.60480935 | <10^-3^ | Minimal |
| 0.5 | B | Brain Size normalized by mass | 0.294358233 | <10^-3^ | Maximal |
| 0.5 | S | Brain Size normalized by mass | 0.090713366 | <10^-3^ | Maximal |
| 0.5 | W | Brain Size normalized by mass | -0.362853352 | <10^-3^ | Maximal |
| 0.5 | W | Female maturity normalized by longevity | -0.005291516 | 0.001 | Maximal |
| 0.05 | S | Gestation/Incubation normalized by longevity | 0.26760624 | <10^-3^ | Maximal |
| 0.1 | B | Gestation/Incubation normalized by longevity | -0.435066942 | <10^-3^ | Minimal |
| 0.1 | S | Gestation/Incubation normalized by longevity | 0.218645604 | <10^-3^ | Maximal |
| 0.1 | W | Gestation/Incubation normalized by longevity | 0.522146173 | <10^-3^ | Maximal |
| 0.2 | B | Gestation/Incubation normalized by longevity | -0.417616658 | <10^-3^ | Minimal |
| 0.2 | W | Gestation/Incubation normalized by longevity | 0.459894853 | <10^-3^ | Maximal |
| 0.5 | B | Gestation/Incubation normalized by longevity | -0.302583824 | <10^-3^ | Maximal |
| 0.5 | W | Gestation/Incubation normalized by longevity | 0.195058262 | <10^-3^ | Maximal |
| 0.05 | W | Inter-litter/Interbirth interval | 0.367126061 | <10^-3^ | Maximal |
| 0.1 | S | Inter-litter/Interbirth interval | -0.846369842 | <10^-3^ | Minimal |
| 0.1 | W | Inter-litter/Interbirth interval | 0.239796393 | <10^-3^ | Maximal |
| 0.2 | S | Inter-litter/Interbirth interval | -0.717194824 | <10^-3^ | Minimal |
| 0.2 | W | Inter-litter/Interbirth interval | 0.091883677 | <10^-3^ | Maximal |
| 0.5 | B | Inter-litter/Interbirth interval | 0 | <10^-3^ | Maximal |
| 0.5 | S | Inter-litter/Interbirth interval | -0.281259497 | <10^-3^ | Maximal |
| 0.5 | W | Inter-litter/Interbirth interval | 0 | <10^-3^ | Maximal |
| 0.005 | B | Litter/Clutch size | -0.397940009 | <10^-3^ | Minimal |
| 0.005 | W | Litter/Clutch size | -0.397940009 | <10^-3^ | Minimal |
| 0.01 | B | Litter/Clutch size | -0.397940009 | <10^-3^ | Minimal |
| 0.01 | W | Litter/Clutch size | -0.397940009 | <10^-3^ | Minimal |
| 0.05 | S | Litter/Clutch size | 0.301029996 | 0.034 | Maximal |
| 0.05 | W | Litter/Clutch size | -0.397940009 | <10^-3^ | Minimal |
| 0.1 | W | Litter/Clutch size | -0.397940009 | <10^-3^ | Minimal |
| 0.2 | B | Litter/Clutch size | 0.079181246 | <10^-3^ | Maximal |
| 0.2 | S | Litter/Clutch size | 0.204119983 | 0.001 | Maximal |
| 0.2 | W | Litter/Clutch size | -0.397940009 | <10^-3^ | Minimal |
| 0.5 | W | Litter/Clutch size | -0.22184875 | <10^-3^ | Maximal |
| 0.05 | B | Litters/Clutches per year | 0 | <10^-3^ | Minimal |
| 0.05 | W | Litters/Clutches per year | -0.301029996 | <10^-3^ | Minimal |
| 0.1 | S | Litters/Clutches per year | 0.301029996 | 0.007 | Maximal |
| 0.1 | W | Litters/Clutches per year | -0.045757491 | <10^-3^ | Minimal |
| 0.2 | S | Litters/Clutches per year | 0.301029996 | <10^-3^ | Maximal |
| 0.5 | B | Litters/Clutches per year | 0 | <10^-3^ | Maximal |
| 0.5 | S | Litters/Clutches per year | 0.176091259 | <10^-3^ | Maximal |
| 0.5 | W | Litters/Clutches per year | 0 | <10^-3^ | Maximal |
| 0.05 | B | Male maturity normalized by longevity | -0.137946865 | <10^-3^ | Minimal |
| 0.1 | B | Male maturity normalized by longevity | -0.093785538 | <10^-3^ | Minimal |
| 0.1 | W | Male maturity normalized by longevity | 0.090031659 | <10^-3^ | Maximal |
| 0.2 | S | Male maturity normalized by longevity | 0.056241304 | 0.017 | Maximal |
| 0.2 | W | Male maturity normalized by longevity | 0.049350208 | <10^-3^ | Maximal |
| 0.5 | W | Male maturity normalized by longevity | -0.005795435 | 0.008 | Maximal |
| 0.01 | W | Metabolic rate normalized by mass | -1.042424919 | <10^-3^ | Minimal |
| 0.05 | W | Metabolic rate normalized by mass | -0.784499774 | <10^-3^ | Minimal |
| 0.1 | W | Metabolic rate normalized by mass | -0.719032917 | <10^-3^ | Minimal |
| 0.2 | B | Metabolic rate normalized by mass | 0.416472783 | <10^-3^ | Maximal |
| 0.2 | S | Metabolic rate normalized by mass | 0.098012157 | <10^-3^ | Maximal |
| 0.2 | W | Metabolic rate normalized by mass | -0.553638109 | <10^-3^ | Minimal |
| 0.5 | B | Metabolic rate normalized by mass | 0.226945831 | <10^-3^ | Maximal |
| 0.5 | S | Metabolic rate normalized by mass | 0.063798679 | 0.011 | Maximal |
| 0.5 | W | Metabolic rate normalized by mass | -0.337699256 | <10^-3^ | Maximal |
| 0.2 | B | Temperature (K) | -0.001262893 | <10^-3^ | Minimal |
| 0.2 | W | Temperature (K) | 0.001539557 | <10^-3^ | Maximal |
| 0.5 | B | Temperature (K) | -0.000420692 | 0.023 | Maximal |
| 0.5 | W | Temperature (K) | 0.000700249 | <10^-3^ | Maximal |
| 0.01 | B | Weaning normalized by longevity | -0.678296857 | <10^-3^ | Minimal |
| 0.05 | B | Weaning normalized by longevity | -0.27808253 | <10^-3^ | Minimal |
| 0.1 | B | Weaning normalized by longevity | -0.219434428 | <10^-3^ | Minimal |
| 0.2 | B | Weaning normalized by longevity | -0.149533089 | <10^-3^ | Minimal |
| 0.2 | W | Weaning normalized by longevity | 0.139559628 | <10^-3^ | Maximal |
| 0.5 | B | Weaning normalized by longevity | -0.073476619 | <10^-3^ | Maximal |
| 0.5 | S | Weaning normalized by longevity | -0.048848568 | <10^-3^ | Maximal |
| 0.5 | W | Weaning normalized by longevity | 0.106849326 | <10^-3^ | Maximal |
| 0.05 | B | Weaning weight normalized by mass | 0.254404784 | <10^-3^ | Maximal |
| 0.1 | B | Weaning weight normalized by mass | 0.235097321 | <10^-3^ | Maximal |
| 0.2 | S | Weaning weight normalized by mass | 0.095132246 | <10^-3^ | Maximal |
| 0.5 | B | Weaning weight normalized by mass | 0.118370831 | <10^-3^ | Maximal |
| 0.5 | S | Weaning weight normalized by mass | 0.078313525 | <10^-3^ | Maximal |
| 0.5 | W | Weaning weight normalized by mass | -0.181463944 | <10^-3^ | Maximal |
