# Supplementary material for: The Mass-Longevity Triangle: Pareto Optimality and the Geometry of Life-History Trait Space
Source: PLoS Comput Biol. 2015 Oct 14;11(10):e1004524. doi: 10.1371/journal.pcbi.1004524 (PMC4605829; doi:10.1371/journal.pcbi.1004524)
Supplement: S2 Table — The first 3 columns show the journal reference of previous studies in which triangles were used to describe the data. The last 4 columns describe how the paper relates to the data, as tasks, traits or characteristic. (DOCX) [file pcbi.1004524.s007.docx]

| **Paper** | **Author, Year** | **Journal** | **Traits** | **Tasks** | **Syndrome/Characteristics** | **Data** |
| --- | --- | --- | --- | --- | --- | --- |
| Evidence for the Existence of Three Primary Strategies in Plants and Its relevance to Ecological and Evolutionary theory [21] | J P Grime, 1977 | The American Naturalist |  | 1. Competitive 2. Stress-tolerant 3. Ruderal | 1. Morphology of shoot 2. Leaf form 3. Litter 4. Life Form 5. Longevity of Leaves 6. Phenology of Flowers 7. Proportion of annual production devoted to seeds | Plants |
| Patterns of Life-History Diversification in North American Fishes: Implications for Population Regulation [41] | Winemiller & Rose, 1992 | Canadian Journal of Fisheries and Aquatic Sciences | 1. Survival (Juvenile survivorship) 2. Fecundity 3. Onset and duration of reproductive life (age of maturity) | 1. Periodic 2. Opportunistic 3. Equilibrium 4. Intermediate | 1. Delayed maturation, large size at maturation, large clutches, small eggs, rapid larval and YOY growth rate, short reproductive season. 2. Early maturation, small size at maturation, small eggs, rapid larval growth rate, long reproductive season with multiple spawning bouts. 3. Small-medium size, small clutches, large eggs, slow YOY and adult growth, long reproductive season, well developed parental care. | Fresh and marine fish |
| Evidence for a three-way trade-off between nitrogen and phosphorus competitive abilities and cell size in phytoplankton [42] | Edwards, Klausmeier & Litchman, 2011 | Ecology | 1. Competititve ability for nitrate 2. Competititve ability for phosphate 3. Cell volume | 1. Competititve ability for nitrate 2. Competititve ability for phosphate 3. Cell volume |  | Phytoplankton |
| Evaluating life history strategies of reef corals from species traits [39] | Darling, Alvarez-Filip, Oliver, McClanahan & Cote, 2012 | Ecology Letters | PCoA of:   1. domed morphology 2. growth rate 3. brooding reproduction 4. fecundity 5. broadcast spawning reproduction 6. branching morphology 7. colony size 8. skeletal density 9. plating morphology 10. corallite diameter 11. depth 12. symbiont diversity 13. generation length 14. solitary colonies | 1. Competititve 2. Stress tolerant 3. Weedy 4. Generalist |  | Reef corals |
| A trait based framework to understand life history on mycorrhizal fungi (opinion) [40] | Chagnon, Bradley, Maherali, Klironomos, 2013 | Trends in plant science |  | 1. Competitive 2. Stress-tolerant 3. Ruderal |  | AM fungi |
