# Supplementary material for: The Mass-Longevity Triangle: Pareto Optimality and the Geometry of Life-History Trait Space
Source: PLoS Comput Biol. 2015 Oct 14;11(10):e1004524. doi: 10.1371/journal.pcbi.1004524 (PMC4605829; doi:10.1371/journal.pcbi.1004524)
Supplement: S3 Table — Here we show the taxonomy of closely related species and their mass and longevity values. (DOCX) [file pcbi.1004524.s008.docx]

**Closely related species that lie far on the mass longevity triangle**

| **Order** | **Family** | **Genus** | **Species** | **Common name** | **Mass (g)** | **Longevity (Yrs)** |
| --- | --- | --- | --- | --- | --- | --- |
| Diprotodontia | Macropodidae | Macropus | bernardus | Black wallaroo | 17500 | 11.8 |
| Diprotodontia | Macropodidae | Onychogalea | unguifera | Northern nail-tailed wallaby | 5800 | 5.5 |
| Artiodactyla | Bovidae | Ovibos | moschatus | Muskox | 315000 | 27.4 |
| Artiodactyla | Cervidae | Blastocerus | dichotomus | Marsh deer | 102500 | 11.17 |
| Primates | Pitheciidae | Pithecia | pithecia | Guianan saki | 1480 | 36 |
| Primates | Callitrichidae | Saguinus | leucopus | Silvery-brown bare-face tamarin | 490 | 15.2 |
| Carnivora | Canidae | Atelocynus | microtis | Small-eared dog | 9500 | 11.9 |
| Carnivora | Canidae | Vulpes | corsac | Corsac fox | 2700 | 13 |
| Chiroptera | Pteropodidae | Rousettus | egyptiacus | Egyptian rousette | 125 | 22.9 |
| Chiroptera | Pteropodidae | Rousettus | leschenaulti | Leschenault's rousette | 108.25 | 14 |
| Diprotodontia | Macropodidae | Petrogale | brachyotis | Western rock wallaby | 3700 | 10.1 |
| Diprotodontia | Macropodidae | Petrogale | concinna | Nabarlek | 1250 | 11.7 |
| Artiodactyla | Bovidae | Gazella | cuvieri | Cuvier's gazelle | 17500 | 17.9 |
| Artiodactyla | Bovidae | Gazella | leptoceros | Rhim gazelle | 48500 | 14.6 |
| Artiodactyla | Cervidae | Blastocerus | dichotomus | Marsh deer | 102500 | 11.17 |
| Artiodactyla | Cervidae | Pudu | puda | Southern pudu | 10000 | 18.3 |
| Chiroptera | Vespertilionidae | Myotis | brandti | Brandt's bat | 7 | 41 |
| Chiroptera | Vespertilionidae | Myotis | nigricans | Black myotis | 4.2 | 7 |
| Chiroptera | Vespertilionidae | Nycticeius | humeralis | Evening bat | 9.75 | 6 |
| Chiroptera | Vespertilionidae | Myotis | mystacinus | Whiskered bat | 5.3 | 24 |
| Artiodactyla | Bovidae | Madoqua | guentheri | Gunther's dik-dik | 4550 | 17.5 |
| Artiodactyla | Bovidae | Bos | taurus | Domestic cattle | 750000 | 20 |
| Dasyuromorphia | Dasyuridae | Dasyurus | maculatus | Australian tiger cat | 5500 | 6.8 |
| Dasyuromorphia | Dasyuridae | Pseudantechinus | macdonnellensis | Fat-tailed marsupial mouse | 32.5 | 7 |
| Chiroptera | Vespertilionidae | Myotis | vivesi | Fish-eating bat | 25 | 10 |
| Chiroptera | Vespertilionidae | Myotis | brandti | Brandt's bat | 7 | 41 |
| Chiroptera | Vespertilionidae | Nycticeius | humeralis | Evening bat | 9.75 | 6 |
| Chiroptera | Vespertilionidae | Plecotus | auritus | Brown big-eared bat | 7.8 | 30 |
| Dasyuromorphia | Dasyuridae | Dasyurus | maculatus | Australian tiger cat | 5500 | 6.8 |
| Dasyuromorphia | Dasyuridae | Sminthopsis | crassicaudata | Fat-tailed dunnart | 15 | 5 |
| Chiroptera | Vespertilionidae | Nycticeius | humeralis | Evening bat | 9.75 | 6 |
| Chiroptera | Vespertilionidae | Myotis | lucifugus | Little brown bat | 10 | 34 |
| Cingulata | Dasypodidae | Priodontes | maximus | Giant armadillo | 33000 | 15 |
| Cingulata | Dasypodidae | Tolypeutes | matacus | La Plata three-banded armadillo | 1500 | 36.8 |
| Dasyuromorphia | Dasyuridae | Dasyurus | maculatus | Australian tiger cat | 5500 | 6.8 |
| Dasyuromorphia | Dasyuridae | Sminthopsis | youngsoni | Lesser hairy-footed dunnart | 10 | 5.3 |
| Dasyuromorphia | Dasyuridae | Dasyurus | maculatus | Australian tiger cat | 5500 | 6.8 |
| Dasyuromorphia | Dasyuridae | Planigale | maculata | Pygmy planigale | 10 | 5.8 |
| Chiroptera | Vespertilionidae | Nycticeius | humeralis | Evening bat | 9.75 | 6 |
| Chiroptera | Vespertilionidae | Myotis | brandti | Brandt's bat | 7 | 41 |
| Dasyuromorphia | Dasyuridae | Dasyurus | maculatus | Australian tiger cat | 5500 | 6.8 |
| Dasyuromorphia | Dasyuridae | Planigale | tenuirostris | Narrow-nosed planigale | 5.3 | 5.2 |
