# Supplementary material for: The Mass-Longevity Triangle: Pareto Optimality and the Geometry of Life-History Trait Space
Source: PLoS Comput Biol. 2015 Oct 14;11(10):e1004524. doi: 10.1371/journal.pcbi.1004524 (PMC4605829; doi:10.1371/journal.pcbi.1004524)
Supplement: S4 Table — Here we show the taxonomy of species and their mass and longevity values. (DOCX) [file pcbi.1004524.s009.docx]

**Far species on the phylogenetic tree that lie close to each other on the mass longevity triangle**

| **Order** | **Family** | **Genus** | **Species** | **Common name** | **Mass (g)** | **Longevity (Yrs)** |
| --- | --- | --- | --- | --- | --- | --- |
| Rodentia | Pedetidae | Pedetes | capensis | Springhare | 3500 | 20 |
| Primates | Indridae | Propithecus | tattersalli | Golden-crowned sifaka | 3545 | 20 |
| Rodentia | Dipodidae | Allactaga | major | Great jerboa | 350 | 7 |
| Rodentia | Echimyidae | Thrichomys | apereoides | Brazilian spiny rat | 373.5 | 7 |
| Rodentia | Dipodidae | Zapus | hudsonius | Meadow jumping mouse | 18 | 5.6 |
| Rodentia | Heteromyidae | Perognathus | parvus | Great Basin pocket mouse | 20.1 | 5.8 |
| Rodentia | Muridae | Mesembriomys | gouldii | Black-footed tree rat | 900 | 7.8 |
| Lagomorpha | Leporidae | Sylvilagus | audubonii | Audubon's cottontail rabbit | 900 | 7.8 |
| Rodentia | Muridae | Zyzomys | palatilis | Carpentarian rock rat | 123 | 6.2 |
| Rodentia | Geomyidae | Thomomys | bottae | Botta's pocket gopher | 115.5 | 6.1 |
| Rodentia | Muridae | Zyzomys | palatilis | Carpentarian rock rat | 123 | 6.2 |
| Rodentia | Myoxidae | Eliomys | melanurus | Asian garden dormouse | 122.5 | 6.2 |
| Rodentia | Muridae | Nyctomys | sumichrasti | Vesper rat | 50 | 5.7 |
| Afrosoricida | Tenrecidae | Microgale | talazaci | Talazaci's long-tailed tenrec | 50 | 5.8 |
| Rodentia | Muridae | Peromyscus | guatemalensis | Guatemalan deer mouse | 40 | 5.7 |
| Afrosoricida | Tenrecidae | Microgale | dobsoni | Dobson's long-tailed tenrec | 37.8 | 5.6 |
| Rodentia | Muridae | Lophiomys | imhausi | Crested rat | 755 | 7.5 |
| Lagomorpha | Leporidae | Sylvilagus | nuttallii | Nuttall's cottontail rabbit | 760 | 7.4 |
| Rodentia | Heteromyidae | Dipodomys | merriami | Merriam's kangaroo rat | 42 | 9.7 |
| Rodentia | Sciuridae | Tamias | minimus | Least chipmunk | 44.1 | 10 |
| Rodentia | Heteromyidae | Dipodomys | deserti | Desert kangaroo rat | 116 | 8.6 |
| Rodentia | Myoxidae | Myoxus | glis | Fat dormouse | 125 | 8.7 |
| Rodentia | Geomyidae | Thomomys | bottae | Botta's pocket gopher | 115.5 | 6.1 |
| Rodentia | Myoxidae | Eliomys | melanurus | Asian garden dormouse | 122.5 | 6.2 |
| Rodentia | Octodontidae | Octodon | degus | Degus | 235 | 14 |
| Afrosoricida | Tenrecidae | Setifer | setosus | Greater hedgehog tenrec | 225 | 14.1 |
| Rodentia | Caviidae | Cavia | porcellus | Guinea pig | 728 | 12 |
| Carnivora | Herpestidae | Galerella | pulverulenta | Cape grey mongoose | 650 | 11.7 |
| Rodentia | Dasyproctidae | Dasyprocta | cristata | Guyanan agouti | 2650 | 14 |
| Carnivora | Felidae | Felis | margarita | Sand cat | 2750 | 13.9 |
| Rodentia | Dasyproctidae | Dasyprocta | mexicana | Mexican agouti | 2700 | 13.1 |
| Lagomorpha | Leporidae | Lepus | brachyurus | Japanese hare | 2519 | 13 |
| Rodentia | Dasyproctidae | Dasyprocta | mexicana | Mexican agouti | 2700 | 13.1 |
| Carnivora | Canidae | Vulpes | corsac | Corsac fox | 2700 | 13 |
| Rodentia | Dasyproctidae | Dasyprocta | mexicana | Mexican agouti | 2700 | 13.1 |
| Hyracoidea | Procaviidae | Dendrohyrax | arboreus | Tree hyrax | 3000 | 13.6 |
| Rodentia | Dasyproctidae | Dasyprocta | prymnolopha | Hairy-rumpted agouti | 2900 | 16.3 |
| Carnivora | Canidae | Vulpes | pallida | Pale fox | 2800 | 16 |
| Rodentia | Caviidae | Dolichotis | patagonum | Patagonian cavy | 12500 | 14.4 |
| Artiodactyla | Cervidae | Hydropotes | inermis | Chinese water deer | 11500 | 13.9 |
| Rodentia | Hystricidae | Hystrix | cristata | African porcupine | 20000 | 28 |
| Artiodactyla | Tayassuidae | Tayassu | pecari | White-lipped peccary | 22000 | 28.5 |
| Rodentia | Hystricidae | Hystrix | brachyura | Old World porcupine | 8000 | 27.3 |
| Primates | Cercopithecidae | Trachypithecus | francoisi | Francois' langur | 7325 | 26.3 |
| Rodentia | Hystricidae | Hystrix | brachyura | Old World porcupine | 8000 | 27.3 |
| Carnivora | Felidae | Leopardus | pardalis | Ocelot | 8800 | 28.2 |
| Rodentia | Bathyergidae | Georychus | capensis | Cape mole-rat | 181 | 11.2 |
| Scandentia | Tupaiidae | Tupaia | tana | Large tree shrew | 197.8 | 11.6 |
| Rodentia | Erethizontidae | Coendou | prehensilis | Prehensile-tailed porcupine | 3900 | 26.6 |
| Carnivora | Procyonidae | Nasua | narica | White-nosed coati | 3750 | 26.4 |
| Rodentia | Erethizontidae | Coendou | prehensilis | Prehensile-tailed porcupine | 3900 | 26.6 |
| Carnivora | Viverridae | Paguma | larvata | Masked palm civet | 4300 | 27.4 |
| Rodentia | Octodontidae | Spalacopus | cyanus | Coruro | 98 | 10.8 |
| Rodentia | Sciuridae | Ammospermophilus | leucurus | White-tailed antelope squirrel | 105 | 11.2 |
| Rodentia | Sciuridae | Marmota | flaviventris | Yellow-bellied marmot | 3500 | 21.2 |
| Carnivora | Herpestidae | Atilax | paludinosus | Marsh mongoose | 3300 | 20.8 |
| Rodentia | Sciuridae | Marmota | caligata | Hoary marmot | 4300 | 12.1 |
| Lagomorpha | Leporidae | Lepus | californicus | Black-tailed jack rabbit | 4175 | 11.8 |
| Rodentia | Sciuridae | Tamias | dorsalis | Cliff chipmunk | 62.8 | 12.6 |
| Scandentia | Tupaiidae | Tupaia | minor | Lesser tree shrew | 58.6 | 12.2 |
| Rodentia | Sciuridae | Tamias | minimus | Least chipmunk | 44.1 | 10 |
| Chiroptera | Pteropodidae | Cynopterus | brachyotis | Lesser short-nosed fruit bat | 45 | 10.1 |
| Rodentia | Sciuridae | Xerus | erythropus | Striped ground squirrel | 600 | 10.3 |
| Carnivora | Mephitidae | Spilogale | putorius | Spotted skunk | 600 | 10.5 |
| Rodentia | Sciuridae | Xerus | inauris | South African ground squirrel | 588 | 11.5 |
| Carnivora | Herpestidae | Galerella | pulverulenta | Cape grey mongoose | 650 | 11.7 |
| Rodentia | Sciuridae | Sciurus | carolinensis | Eastern gray squirrel | 533 | 23.6 |
| Primates | Callitrichidae | Saguinus | imperator | Emperor tamarin | 518.5 | 23.7 |
| Rodentia | Sciuridae | Sciurus | niger | Eastern fox squirrel | 800 | 16 |
| Cingulata | Dasypodidae | Chaetophractus | vellerosus | Leeser hairy armadillo | 840 | 16.2 |
| Rodentia | Sciuridae | Sciurus | granatensis | Red-tailed squirrel | 374 | 11.5 |
| Scandentia | Tupaiidae | Urogale | everetti | Philippine tree shrew | 350 | 11.5 |
| Rodentia | Sciuridae | Sciurus | variegatoides | Variegated squirrel | 485 | 15.1 |
| Primates | Callitrichidae | Saguinus | leucopus | Silvery-brown bare-face tamarin | 490 | 15.2 |
| Rodentia | Sciuridae | Sciurus | aureogaster | Red-bellied squirrel | 600 | 11.5 |
| Carnivora | Herpestidae | Galerella | pulverulenta | Cape grey mongoose | 650 | 11.7 |
| Rodentia | Sciuridae | Sundasciurus | tenuis | Slender squirrel | 75 | 10 |
| Chiroptera | Pteropodidae | Cynopterus | sphinx | Greater short-nosed fruit bat | 75 | 10 |
| Rodentia | Sciuridae | Sundasciurus | tenuis | Slender squirrel | 75 | 10 |
| Chiroptera | Pteropodidae | Epomophorus | wahlbergi | Wahlberg's epauletted fruit bat | 80 | 10.1 |
| Lagomorpha | Leporidae | Lepus | brachyurus | Japanese hare | 2519 | 13 |
| Carnivora | Mustelidae | Galictis | vittata | Grison | 2300 | 12.5 |
| Primates | Cercopithecidae | Cercopithecus | cephus | Moustached monkey | 3585 | 36 |
| Primates | Lemuridae | Varecia | variegata | Ruffed lemur | 3670 | 37 |
| Primates | Cercopithecidae | Cercopithecus | mona | Mona monkey | 4500 | 30 |
| Primates | Indridae | Propithecus | verreauxi | Verreaux's sifaka | 5000 | 31 |
| Primates | Cercopithecidae | Cercopithecus | lhoesti | L'hoest's monkey | 4700 | 24.1 |
| Carnivora | Procyonidae | Nasua | nasua | Coatimundi | 4750 | 23.7 |
| Primates | Cercopithecidae | Miopithecus | talapoin | Talapoin | 1384.5 | 27.7 |
| Primates | Loridae | Perodicticus | potto | Potto | 1225 | 26.8 |
| Primates | Cercopithecidae | Macaca | thibetana | Tibetan macaque | 10300 | 27 |
| Carnivora | Felidae | Lynx | canadensis | Canada lynx | 10100 | 26.8 |
| Primates | Cercopithecidae | Trachypithecus | auratus | Javan langur | 9720 | 31.1 |
| Carnivora | Mustelidae | Mellivora | capensis | Honey badger | 10000 | 31.3 |
| Primates | Cercopithecidae | Trachypithecus | pileatus | Capped leaf monkey | 11450 | 25.2 |
| Artiodactyla | Bovidae | Oreotragus | oreotragus | Klipspringer | 12000 | 25.9 |
| Primates | Cercopithecidae | Trachypithecus | phayrei | Phayre's leaf monkey | 8400 | 28.3 |
| Carnivora | Felidae | Leopardus | pardalis | Ocelot | 8800 | 28.2 |
| Primates | Cercopithecidae | Pygathrix | nemaeus | Douc langur | 9720 | 26 |
| Carnivora | Felidae | Lynx | canadensis | Canada lynx | 10100 | 26.8 |
| Primates | Pitheciidae | Cacajao | calvus | Red uakari | 3165 | 35.8 |
| Primates | Lemuridae | Eulemur | fulvus | Brown lemur | 3150 | 35.5 |
| Primates | Pitheciidae | Pithecia | pithecia | Guianan saki | 1480 | 36 |
| Cingulata | Dasypodidae | Tolypeutes | matacus | La Plata three-banded armadillo | 1500 | 36.8 |
| Primates | Pitheciidae | Callicebus | moloch | Dusky titi | 804 | 26.2 |
| Carnivora | Eupleridae | Galidia | elegans | Malagasy ring-tailed mongoose | 800 | 26 |
| Primates | Pitheciidae | Callicebus | cupreus | Coppery titi | 1120 | 26.4 |
| Primates | Loridae | Perodicticus | potto | Potto | 1225 | 26.8 |
| Primates | Pitheciidae | Callicebus | donacophilus | Bolivian titi | 795 | 25 |
| Primates | Loridae | Nycticebus | coucang | Slow loris | 890.5 | 25.8 |
| Primates | Lemuridae | Hapalemur | simus | Greater bamboo lemur | 1737.5 | 17.6 |
| Carnivora | Herpestidae | Herpestes | smithii | Ruddy mongoose | 1702.5 | 17.8 |
| Primates | Galagonidae | Galago | senegalensis | Senegal galago | 192.2 | 17.1 |
| Chiroptera | Pteropodidae | Pteropus | pumilus | Little golden-mantled flying fox | 200 | 17.2 |
| Primates | Galagonidae | Otolemur | garnettii | Small-eared galago | 1300 | 18.3 |
| Carnivora | Mustelidae | Martes | martes | European pine marten | 1300 | 18.2 |
| Artiodactyla | Bovidae | Oryx | dammah | Scimitar-horned oryx | 177500 | 27.5 |
| Carnivora | Felidae | Panthera | leo | Lion | 175000 | 27 |
| Artiodactyla | Bovidae | Capra | cylindricornis | East Caucasian tur | 50000 | 20.2 |
| Carnivora | Felidae | Acinonyx | jubatus | Cheetah | 53500 | 20.5 |
| Artiodactyla | Bovidae | Capra | nubiana | Nubian ibex | 46250 | 22.4 |
| Carnivora | Otariidae | Arctocephalus | tropicalis | Sub-Antarctic fur seal | 50000 | 23 |
| Artiodactyla | Bovidae | Pseudois | nayaur | Bharal | 55000 | 20.9 |
| Carnivora | Felidae | Acinonyx | jubatus | Cheetah | 53500 | 20.5 |
| Artiodactyla | Bovidae | Naemorhedus | caudatus | Chinese goral | 27000 | 20.3 |
| Carnivora | Canidae | Canis | lupus | Gray wolf | 26625 | 20.6 |
| Artiodactyla | Bovidae | Gazella | dorcas | Dorcas gazelle | 14500 | 23.7 |
| Carnivora | Felidae | Catopuma | temminckii | Asiatic golden cat | 13500 | 23 |
| Artiodactyla | Bovidae | Cephalophus | dorsalis | Bay duiker | 11600 | 17.5 |
| Carnivora | Felidae | Prionailurus | viverrinus | Fishing cat | 10650 | 17.2 |
| Artiodactyla | Bovidae | Madoqua | guentheri | Gunther's dik-dik | 4550 | 17.5 |
| Carnivora | Canidae | Otocyon | megalotis | Bat-eared fox | 4150 | 17 |
| Artiodactyla | Bovidae | Madoqua | kirkii | Kirk's dik-dik | 5000 | 17.3 |
| Carnivora | Felidae | Prionailurus | bengalensis | Leopard cat | 5000 | 17 |
| Artiodactyla | Cervidae | Pudu | puda | Southern pudu | 10000 | 18.3 |
| Carnivora | Canidae | Canis | aureus | Golden jackal | 11000 | 18.8 |
| Artiodactyla | Cervidae | Elaphurus | davidianus | Pere David's deer | 186500 | 27.5 |
| Carnivora | Felidae | Panthera | leo | Lion | 175000 | 27 |
| Artiodactyla | Tragulidae | Tragulus | javanicus | Lesser mouse-deer | 2850 | 14 |
| Carnivora | Mustelidae | Martes | pennanti | Fisher | 3175 | 14.3 |
| Artiodactyla | Tragulidae | Tragulus | javanicus | Lesser mouse-deer | 2850 | 14 |
| Carnivora | Felidae | Felis | margarita | Sand cat | 2750 | 13.9 |
| Artiodactyla | Tragulidae | Tragulus | napu | Greater mouse-deer | 6500 | 16.7 |
| Carnivora | Canidae | Nyctereutes | procyonoides | Raccoon dog | 6500 | 16.6 |
| Cetacea | Phocoenidae | Phocoena | phocoena | Harbor porpoise | 52500 | 20.4 |
| Carnivora | Felidae | Acinonyx | jubatus | Cheetah | 53500 | 20.5 |
| Cetacea | Delphinidae | Lagenorhynchus | obliquidens | Pacific white-sided dolphin | 103000 | 46 |
| Carnivora | Phocidae | Phoca | vitulina | Harbor seal | 115000 | 47.6 |
| Cetacea | Delphinidae | Grampus | griseus | Risso's dolphin | 425000 | 42.5 |
| Carnivora | Ursidae | Ursus | maritimus | Polar bear | 475000 | 43.8 |
| Cetacea | Delphinidae | Sousa | chinensis | Indo-Pacific humpbacked dolphin | 280000 | 40 |
| Carnivora | Ursidae | Ursus | arctos | Brown bear and grizzly bear | 277500 | 40 |
| Artiodactyla | Suidae | Sus | scrofa | Wild boar | 130000 | 27 |
| Carnivora | Felidae | Panthera | tigris | Tiger | 119700 | 26.3 |
| Carnivora | Mustelidae | Mustela | nivalis | Common weasel | 46.9 | 9.1 |
| Chiroptera | Phyllostomidae | Phyllostomus | discolor | Pale spear-nosed bat | 43.4 | 9 |
| Carnivora | Mustelidae | Mustela | nivalis | Common weasel | 46.9 | 9.1 |
| Macroscelidea | Macroscelididae | Elephantulus | intufi | Long-eared elephant shrew | 52 | 9.3 |
| Carnivora | Ailuridae | Ailurus | fulgens | Lesser panda | 4325 | 19 |
| Pilosa | Myrmecophagidae | Tamandua | tetradactyla | Southern tamandua | 4500 | 19 |
| Carnivora | Canidae | Vulpes | rueppellii | Ruppell's sand fox | 3200 | 14.3 |
| Hyracoidea | Procaviidae | Procavia | capensis | Rock hyrax | 3600 | 14.8 |
| Carnivora | Canidae | Urocyon | cinereoargenteus | Gray fox | 4750 | 16.2 |
| Pilosa | Myrmecophagidae | Tamandua | mexicana | Northern tamandua | 4500 | 16 |
| Carnivora | Herpestidae | Ichneumia | albicauda | White-tailed mongoose | 3500 | 14.8 |
| Hyracoidea | Procaviidae | Procavia | capensis | Rock hyrax | 3600 | 14.8 |
| Chiroptera | Phyllostomidae | Phyllostomus | discolor | Pale spear-nosed bat | 43.4 | 9 |
| Macroscelidea | Macroscelididae | Macroscelides | proboscideus | Short-eared elephant shrew | 40 | 8.7 |
| Monotremata | Tachyglossidae | Zaglossus | bruijni | Long-nosed echidna | 7500 | 41.2 |
| Pilosa | Megalonychidae | Choloepus | didactylus | Linne's two-toed sloth | 6250 | 36.8 |
| Monotremata | Ornithorhynchidae | Ornithorhynchus | anatinus | Duck-billed platypus | 1250 | 22.6 |
| Chiroptera | Pteropodidae | Pteropus | vampyrus | Large flying fox | 872 | 20.9 |
| Monotremata | Tachyglossidae | Tachyglossus | aculeatus | Short-nosed echidna | 3500 | 49.5 |
| Primates | Cebidae | Cebus | olivaceus | Weeping capuchin | 2552.5 | 47 |
| Monotremata | Ornithorhynchidae | Ornithorhynchus | anatinus | Duck-billed platypus | 1250 | 22.6 |
| Primates | Pitheciidae | Pithecia | monachus | Monk saki | 1780 | 25 |
| Monotremata | Tachyglossidae | Zaglossus | bruijni | Long-nosed echidna | 7500 | 41.2 |
| Primates | Cercopithecidae | Cercocebus | torquatus | Red-capped mangabey | 9492.5 | 46 |
| Monotremata | Tachyglossidae | Zaglossus | bruijni | Long-nosed echidna | 7500 | 41.2 |
| Primates | Hylobatidae | Hylobates | klossii | Kloss's gibbon | 5900 | 37 |
| Monotremata | Tachyglossidae | Zaglossus | bruijni | Long-nosed echidna | 7500 | 41.2 |
| Primates | Cercopithecidae | Macaca | mulatta | Rhesus monkey | 8235 | 40 |
| Monotremata | Tachyglossidae | Zaglossus | bruijni | Long-nosed echidna | 7500 | 41.2 |
| Pilosa | Megalonychidae | Choloepus | hoffmanni | Hoffmann's two-toed sloth | 6250 | 41 |
| Monotremata | Tachyglossidae | Tachyglossus | aculeatus | Short-nosed echidna | 3500 | 49.5 |
| Primates | Cebidae | Cebus | apella | Brown capuchin | 2642.5 | 46 |
| Monotremata | Tachyglossidae | Zaglossus | bruijni | Long-nosed echidna | 7500 | 41.2 |
| Primates | Hylobatidae | Hylobates | pileatus | Pileated gibbon | 5735 | 38 |
| Monotremata | Ornithorhynchidae | Ornithorhynchus | anatinus | Duck-billed platypus | 1250 | 22.6 |
| Primates | Galagonidae | Otolemur | crassicaudatus | Greater galago | 1094.5 | 22.7 |
| Monotremata | Tachyglossidae | Zaglossus | bruijni | Long-nosed echidna | 7500 | 41.2 |
| Primates | Cercopithecidae | Macaca | silenus | Liontail macaque | 7875 | 40 |
| Monotremata | Ornithorhynchidae | Ornithorhynchus | anatinus | Duck-billed platypus | 1250 | 22.6 |
| Carnivora | Procyonidae | Bassaricyon | gabbii | Olingo | 1235 | 21.8 |
| Monotremata | Tachyglossidae | Zaglossus | bruijni | Long-nosed echidna | 7500 | 41.2 |
| Primates | Cercopithecidae | Macaca | fascicularis | Long-tailed macaque | 6362.5 | 39 |
| Monotremata | Tachyglossidae | Zaglossus | bruijni | Long-nosed echidna | 7500 | 41.2 |
| Primates | Hylobatidae | Hylobates | hoolock | Hoolock gibbon | 6875 | 41 |
| Monotremata | Ornithorhynchidae | Ornithorhynchus | anatinus | Duck-billed platypus | 1250 | 22.6 |
| Primates | Lemuridae | Hapalemur | griseus | Bamboo lemur | 1347.5 | 23.3 |
